# Optimizing scheduling in dual-pulse nucleoside labeling experiments for cell-cycle analysis

**DOI:** 10.1016/j.bpj.2026.03.049

**Published:** 2026-03-27

**Authors:** Alastar Phelan, Constandina Pospori, Cristina Lo Celso, Chiu Fan Lee

**Affiliations:** 1 Department of Bioengineering, Imperial College London, London, UK; 2 Department of Life Sciences, Imperial College London, London, UK; 3 The Francis Crick Institute, London, UK

## Abstract

All eukaryotic cells go through a universal sequence of phases during their division cycle, where the phase timings vary according to cell type and state. Dual-pulse nucleoside labeling (DPNL) is a standard, widely applicable experimental DNA base-substituting technique to probe cell-cycle kinetics at the population level, including in living organisms. In such an experimental protocol, a key scheduling parameter is the choice of waiting time between the two labeling pulses. Here, we model population cell-cycle dynamics as a three-stage Poisson process with an idealized S-phase labeling step and use a simulation-based look-up procedure to demonstrate that the inter-pulse waiting time can be optimized to maximize the signal-to-noise ratio of inferred cycle parameters—an issue that is especially critical in DPNL experiments with limited cell numbers and replicates. An optimal choice of pulse scheduling typically improves S-phase time inference by 50% compared to a suboptimal choice. We further discuss the procedure to perform such a task in an experimentally relevant setting.

## Significance

Dual-pulse nucleoside labeling (DPNL) is a widely used experimental technique for measuring cell-cycle dynamics, yet a key control parameter—the waiting time between labeling pulses—is typically chosen heuristically. Using a minimal stochastic model of cell-cycle progression, we show that inference accuracy depends non-monotonically on this waiting time and that an optimal pulse separation generically exists. Selecting this optimal timing can improve the signal-to-noise ratio of S-phase inference by up to 50% without increasing cell numbers or experimental repeats. Our results demonstrate that experimental scheduling is a critical and tunable component of quantitative inference in DPNL assays and provide a practical, model-guided framework for improving precision under experimentally realistic constraints.

## Introduction

Tightly regulated cell divisions are crucial to the development and maintenance of all organisms. Before a cell can divide, it must go through multiple phases with carefully controlled checkpoints termed the G_1_ (first gap phase), S (the synthesis phase when DNA is replicated), G_2_ (second gap phase), and M (for mitosis). A quantitative understanding of how much time a cell spends in these distinct phases is fundamental to our understanding of basic cellular functions, from DNA replication to organelle duplication.

**Figure 1:**
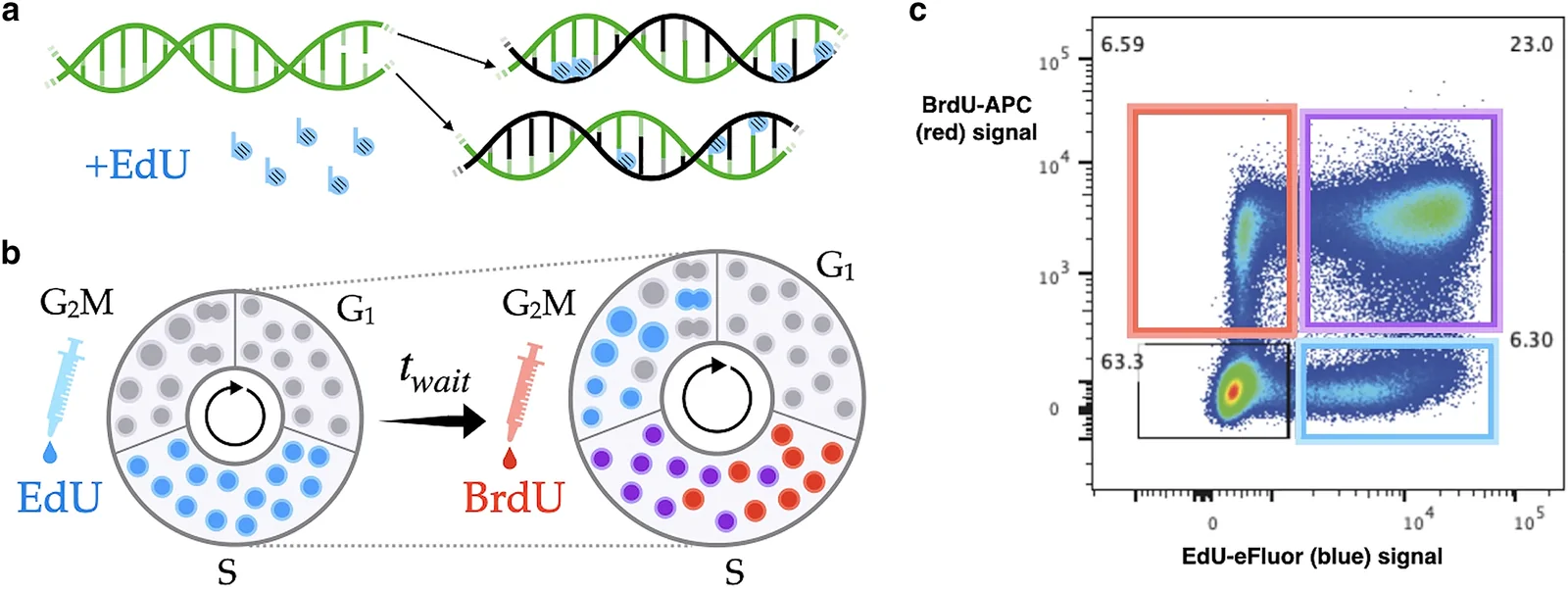
Nucleoside analog incorporation in dual-pulse nucleoside labeling (DPNL) to study the cell cycle. (a) If thymidine analog EdU (5-ethynyl-2′-deoxyuridine) is available during DNA synthesis, it is incorporated into each new half-strand produced. (b) In DPNL, the progress of cells through the cell cycle can be tracked by using a pulse of EdU (blue) followed by BrdU (5-bromo-2′-deoxyuridine, red) to show which cells have finished (blue), remained in (purple), or newly begun (red), DNA synthesis in the time between pulses. (c) The typical output of a dual-pulse labeling assay is a square-shaped distribution of fluorescence intensity, as measured by flow cytometry, which is proportional to the number of analog molecules. There are four density peaks at the coarsest level (3,4) corresponding to the colorings shown in (b). The percentage of cells with each fluorescence combination are shown in text near to each box. The data shown are from bone marrow-situated acute myeloid leukemia cells collected according to the method described in Akinduro et al. (5), with a 2-h waiting time.

Given the importance of understanding cell-cycle dynamics, diverse experimental techniques have been developed to quantify the process (1) and among these the use of nucleoside substitution labeling is particularly popular due to its ease of use and high sensitivity (2). In such an experimental procedure, cells replicating their DNA in the S phase will incorporate modified nucleosides (e.g., 5-ethynyl-2′-deoxyuridine [EdU], a thymidine analog, Fig. 1 *a*) whose presence can then later be detected (e.g., via flow-cytometry methods). Detecting the amount of cells with these modified nucleosides in an EdU pulse-chase experiment has been a gold-standard approach to quantifying the duration they spend in the S phase (6).

To directly probe cell-cycle dynamics, a sequential substitution of nucleosides using two distinct labels (e.g., EdU and 5-bromo-2′-deoxyuridine [BrdU]), termed dual-pulse nucleoside labeling (DPNL), has been developed (7,8). In this experimental procedure, the second label is introduced into the system at a time $t_{\mathrm{wait}}$ after the introduction of the first label (Fig. 1 *b*). As a result, four distinctly labeled cell groups (Fig. 1 *c*) can be detected (as opposed to two in the single-labeling method), boosting the information output of the experiment and especially improving S-phase inference accuracy (9). Double-positive cells (purple, Fig. 1 *b* and *c*) are those which were in S phase during both EdU and BrdU exposure, and single-positive cells (blue, red in Fig. 1 *b* and *c*) are those which were only in S phase at either the EdU or the BrdU exposure time, meaning they were late S phase or early S phase, respectively, at the corresponding pulse time; otherwise, cells are double negative. For populations engaged in a synchronized cell cycle (10), only one of these four label states would be observed in a DPNL assay. While BrdU positivity at the end of a DPNL experiment implies that a cell is in S phase, both the EdU-single-positive and the double-negative populations are split between the G_2_M and G_1_ phases in continuously proliferating cell types. This output structure suggests that S-phase inference will be better constrained by DPNL data compared to G_1_ and G_2_M.

**Figure 2:**
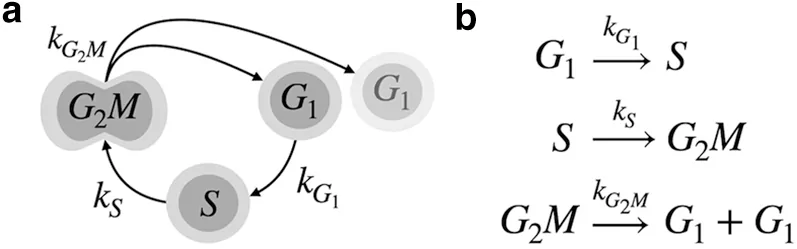
The three-species, three-parameter model of the cell cycle we have used in this work. (a) Cells in each stage i of the cycle advance to the next at a rate $k_{i}$, as a Poisson process. Two new cells are generated at the end of the cycle, both of which continue to advance through the cycle phases from the beginning, G_1_ phase. (b) Reaction scheme describing the cell-cycle phase transitions. As a three-stage Poisson process, the total duration of the cycle is Erlang-distributed (16), while using the minimum number of parameters to keep results fully interpretable.

However, cells *in vivo* are rarely synchronized, and variability (even purely at the population level) in the times that cells within a population take to complete each phase, due to constraints such as tissue crowding (5,11) or mitogenic factors (12,13), remain a challenge for quantitative modeling where detailed timeseries data are scarce (14). Noisy cycle dynamics can have a large impact on inference about a whole population when the number of specific cells of interest (e.g., actively proliferating hematopoietic stem cells in a mouse) may be fewer than 1,000 cells (5). Compounding with this, the number of repeats of cells or tissues sampled is typically small in a given study, highlighting the need to optimize the accuracy of the experimental measurements. Indeed, a clear control parameter in this dual-labeling method is the waiting time $t_{\mathrm{wait}}$ between the introductions of the two labels. A longer $t_{\mathrm{wait}}$ allows more EdU-positive cells to leave S phase before the BrdU pulse, giving a smaller double-positive population, but larger single-positive populations, for $t_{\mathrm{wait}}$ shorter than S phase. However, how to choose $t_{\mathrm{wait}}$ to optimize the information output has thus far, to the best of our knowledge, not been thoroughly investigated. The typical constraints on $t_{\mathrm{wait}}$ are that it must be longer than the timescale for a pulse’s nucleoside analogs to be incorporated into nascent DNA and shorter than the duration of S phase such that there is a double-labeled population from which to infer dynamic information. This can lead to timings from 1.5 to 3 h (15). Two hours is a common choice of $t_{\mathrm{wait}}$ due to the practicality of performing repeat experiments with this timing during a working day, without it being so short that single-labeled populations are too scarce to be reliable to infer dynamic parameters. Here, we perform this task and demonstrate, using simulation of a simple model of cell cycle (Fig. 2), how to find the $t_{\mathrm{wait}}$ that optimizes the signal-to-noise ratio (SNR) of the experimental measurements. Our work provides a proof of principle in how to use modeling to improve the precision of a widely used experimental method in the study of cell-cycle dynamics.

## Materials and methods

### Population-level cell-cycle progression model

We model the progress of the cell cycle through distinct phases equivalently to chemical reactions with rates $k_{i}$ (Fig. 2), subject to fluctuations in the reaction kinetics. We have used a common simplification in the model, namely contracting G_2_ and M phases into a single phase, termed G_2_M. The two phases, usually the shortest in the cycle, are not readily distinguishable when looking solely at EdU fluorescence or DNA content (1,17), hence the phases are often modeled as one.

Cells are modeled as progressing through the cycle asynchronously, which is accepted for quantifying the cell cycle across a cell population in most cases (1,2,10). The model’s strength is in its simplicity, where any other noisy cell-cycle model is necessarily more complex (18,19). The output of pure DPNL has two channels, giving four measurable quantities—the total number of cells with each fluorescence combination (Fig. 1 *b* and *c*)—meaning that any attempt to infer more than four independent model parameters from the output is likely to be difficult.

To find the model parameter-dependent statistics of the labeled cell counts at the end of DPNL experiments so that we can investigate how noise can be mitigated in the process, we model DPNL at the population level stochastically. The Master equation describing probabilistic trajectories of the population state, described by a vector $N={\left(N_{G_{1}},N_{S},N_{G_{2}M}\right)}^{T}$, the number of cells in G_1_, S and G_2_M phase, respectively, is as follows:

(1) $$\frac{dP}{dt}\left(N,t\right)=\overset{3}{\underset{i=1}{\sum }}W_{i}\left(N-\delta N_{i}\right)P\left(N-\delta N_{i},t\right)-W_{i}\left(N\right)P\left(N,t\right)$$

where P(N,t) is the probability at time t of having a population state N. $W_{i}\left(N\right)=k_{i}N_{i}$ is the transition rate out of the state N via a single cell transition $\delta N_{i}$, the loss of one cell and the gain of one or two in the next phase as described in the reaction scheme Fig. 2*b*, with $N_{i=1}$ corresponding to the number of G_1_-phase cells, $k_{i=1}$ being their rate of progression to S phase, and so on for i=2,3.

### Idealized S-phase labeling during the cell cycle

For the labeling part of the model, we apply an EdU label to all cells in the S phase at time t=0 only and apply BrdU to all cells in S phase and either EdU-positive or -negative at $t=t_{\mathrm{wait}}$ only. In other words, labeling is assumed to be instantaneous and complete, as supported experimentally (5). We do not account for EdU dilution after cell division when analyzing flow-cytometry data, as the threshold for EdU positivity can be chosen while taking the dilution into account, with even the most diluted cells readily distinguished from EdU-negative cells. All labeled cells continue to advance through the cell cycle at unaltered rates until the end of the DPNL process, ultimately generating a separately cycling population for each label combination by this time.

Given the chemical master equations and labeling scheme above, we will now analytically calculate the results of an array of DPNL experiments.

### Generating a look-up table from kinetic rates to DPNL outputs

As we focus here on population noise, which manifests itself most strongly in the study of small cell colonies, e.g., actively proliferating stem cells, we initialize our system with 300 cells, consistent with the reported numbers of hematopoietic stem cells entering S phase per hour in the mouse hind leg (5), as an example of a small population of replicating cells. When bulk measurements are made on more abundant cell types, the collected numbers of cells can reach tens of thousands, where the need for stringent optimization is smaller due to reduced relative population noise. The initial composition corresponds to the number of cells in each phase in the steady growth solution from solving for the dynamics of the deterministic model specified in Fig. 2 (see section SM1 of the supporting material for details). Further motivated by the typical experimental procedure (5,15,20), we have a 30-min interval after BrdU administration before counting up the cells with each label combination, which ensured the complete incorporation of BrdU in real experiments.

The total number of cells with each of the four label combinations—EdU single-positive (blue), BrdU single-positive (red), double-positive (purple), and double-negative (gray)—is the output of an experiment (see Fig. 1 *b* and *c*), which can be used to calculate individual phase durations, with the exact calculation depending on the cell type (7,15,18,21,22).

To probe the statistics of labeled cell outputs, we sweep across a range of scenarios by solving analytically (see section SM2 for details) across a grid of model parameters $k_{i}$ and $t_{\mathrm{wait}}$, while fixing the overall cell-cycle length to 24 h. This focuses the analysis on determining the lengths of each phase within a known total period, from which the results can be scaled proportionally to any other cycle length. The parameter sweep range considered (details in section SM3) is within the experimentally relevant range based on observed ratios between G_1_-, S-, and G_2_M-phase duration (Table S1 in Greenberg and Simon (23)) and keeping $t_{\mathrm{wait}}\leq 12$ hours. Beyond this limit, a significant number of cells with a 24-h cycle could both exit S phase after receiving the EdU pulse and re-enter it in another lap of the cycle by the time of the BrdU pulse. Our analysis focuses on continuously proliferating cell populations, for which experimental protocols typically employ short interpulse intervals in order to mitigate this re-entry effect. In rapidly cycling systems, such as certain hematopoietic progenitor populations, the transit through G_2_, M, and G_1_ can in principle occur within a few hours, particularly under stimulatory conditions (24,25). Consequently, pulse separations on the order of 1–2 h are commonly adopted in practice to reduce the likelihood that cells labeled in the first pulse complete a full cycle and re-enter S phase before the second pulse. These cells could make cell-count outputs difficult to distinguish from a slower case where the same number of cells have remained in S phase throughout to register as double positive.

For each combination of model parameters, $k_{i}$ and control parameter $t_{\mathrm{wait}}$, we generate the mean and standard deviation of its output cell numbers’ probability distributions through analytically solving the Master equation (Equation 1) using the standard method described in section SM2. This data set generated will serve as a ground-truth look-up table when we deal with typical experimental studies where the small number of repeats focuses our consideration on the high proportional noise in realistic measurements.

### Inferring cell-cycle transition rates from typical experiments

We now use the ground-truth dataset generated as a dictionary to enable us to perform the inference of the model parameters $k_{i}$ from the outputs of a typical experiment, which consists of only around 3–5 repeats. Hence, we perform 3-repeat simulations using a Gillespie algorithm (26) (see section SM4 for details) for each model parameter combination. For every 3-repeat trial, we look up the closest entry (see section SM3 for details) in the dictionary data previously collected to infer the most likely $k_{i}$. To obtain the statistics of this inference process, we perform a large number $\left(∼10^{3}\right)$ of 3-repeat trials of simulations per model parameter combination to enable us to build up a distribution of estimated $k_{i}$ from the simulated 3-repeat trials. Investigating these distributions will enable us to decipher the SNR of our inference process as a function of the waiting time $t_{\mathrm{wait}}$.

## Results and discussion

In a typical experiment, since the number of repeats is small, the inferred parameters $k_{i}$ will deviate from the true cell-cycle rates due to intrinsic fluctuations.

Consider counting the number of cells in a population before and after a growth period, which is controlled by the experimenter, in order to infer the population’s growth rate per cell. Waiting as long as possible is clearly the ideal case, as the expected deviation of the average division time of a cell is suppressed with longer growth times in line with the central limit theorem. However, in DPNL, the cell-cycle rate parameter inference cannot keep improving for longer waiting times because relatively fast-cycling EdU-positive cells re-entering S phase for the BrdU pulse can be difficult to distinguish from slow-cycling cells that remained in S during that interval, increasing inference error and parameter sensitivity (5). Our simulation approach can quantify this trade-off.

To understand the impact of the stochasticity, we can use the inference SNR:

(2) $$SNR\left\{k_{i}\right\}=\frac{k_{i}}{\sigma_{k_{i}}}$$

which corresponds to the ratio of the average most-likely k values and their standard deviations.

Here, we can quantify the SNRs accurately by comparing results from our 3-repeat *in silico* experiment with the known ground-truth rate parameters in our look-up.

**Figure 3:**
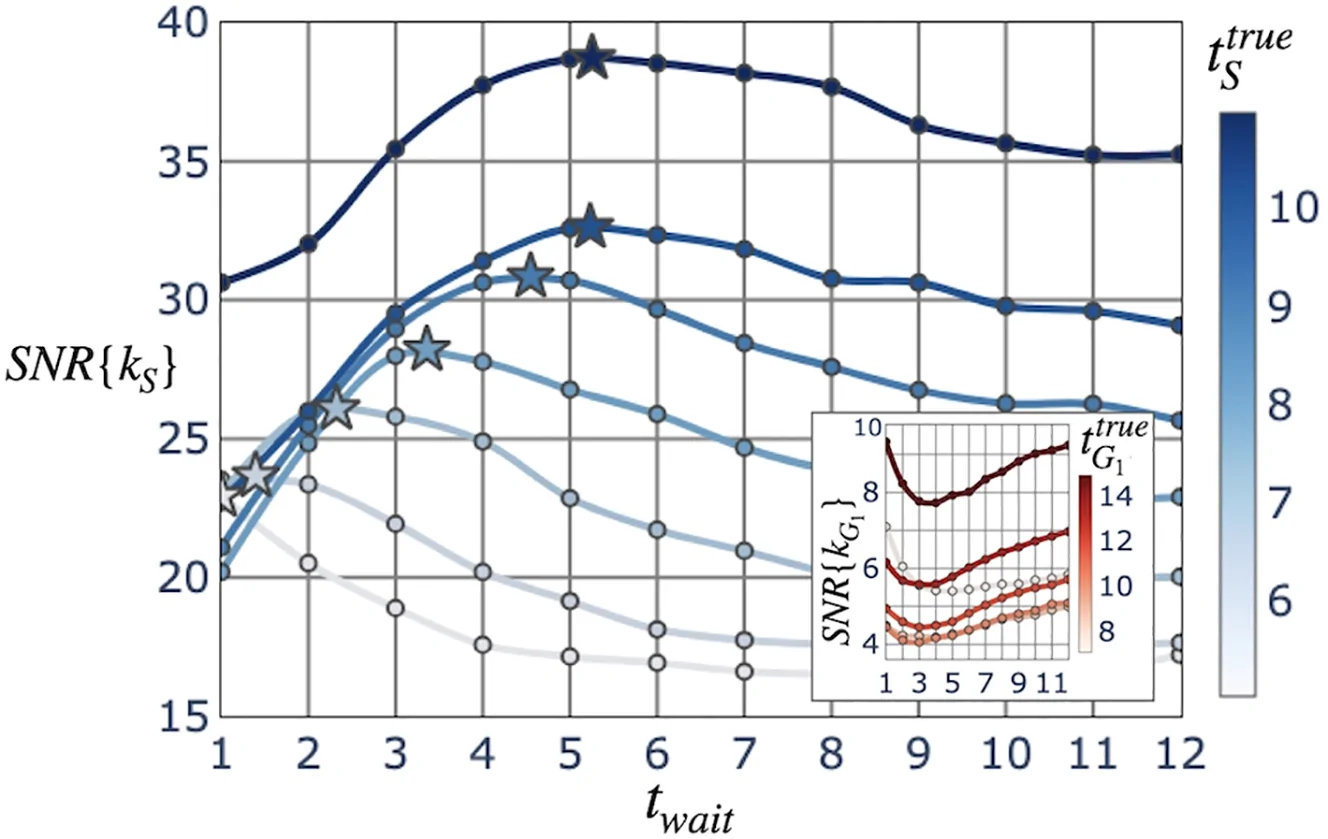
Signal-to-noise ratio (SNR) of the look-up inference process for model parameters $k_{i}$. Main panel: $SNR\left\{k_{S}\right\}$ versus $t_{\mathrm{wait}}$ (in hours) for different S-phase durations (color scale, in hours). A nontrivial S-phase-time-dependent optimum exists, reaching higher peak values for longer S-phase durations, with the optimal pulse interval simultaneously becoming longer. Inset: intermediate pulse interval times are unfavorable for G_1_-phase duration inference across values of *t*_G_1__ (color scale, hours) and hint at an indirect trade-off with S-phase inference. The relative mean-squared inference errors for each parameter are shown in section SM5.

In Fig. 3, we show the SNRs as a function of the waiting time $t_{\mathrm{wait}}$ for distinct set values of S-phase and G_1_-phase periods, $t_{S}$ (Fig. 3, main plot) and $t_{G_{1}}$ (Fig. 3, inset), respectively. We find that *for any* set of initial ground-truth parameters (color bar in Fig. 3), $SNR\left\{k_{S}\right\}$ is generically non-monotonic with respect to $t_{\mathrm{wait}}$, namely, there is indeed an optimal $SNR\left\{k_{S}\right\}$ at a nontrivial value of $t_{\mathrm{wait}}$. Although $SNR\left\{k_{G_{1}}\right\}$ seems to show opposite trends to $SNR\left\{k_{S}\right\}$, the local minima in $SNR\left\{k_{G_{1}}\right\}$ do not coincide with the local maxima of $SNR\left\{k_{S}\right\}$, thus indicating that there exists generally an optimal $t_{\mathrm{wait}}$ that can optimize the SNR of the inference process, depending on the experimental focus (e.g., whether it is on $t_{S}$ or on $t_{G_{1}}$). The peak position is robust to reasonable initial noise and more tightly controlled dwell times in each phase (see section SM6 for details).

**Figure 4:**
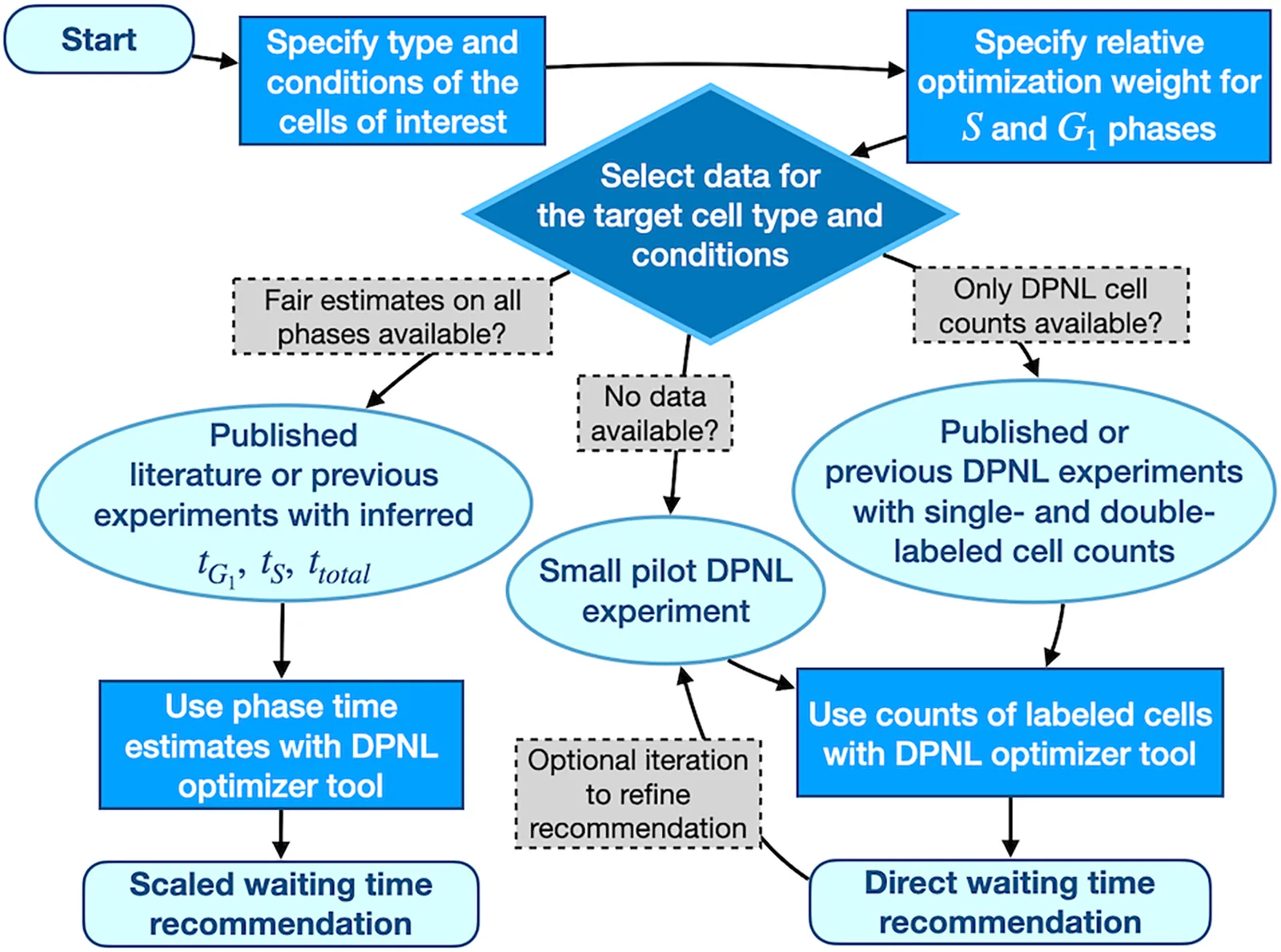
Workflow diagram illustrating optimization of cell-cycle measurement via our look-up-based tool. The relative optimization weights for S and G_1_ phases should be specified when the optimizer tool is called, but prioritizing S phase is advised for a pure DPNL experiment with no supporting measurements that distinguish early and late cells within the cycle in order to separately count G_1_- and G_2_M-phase cells.

We will now describe how such an optimization can be applied in a typical experiment, also shown in Fig. 4.

### A protocol for optimizing the waiting time

(1) Obtain preliminary reference DPNL labeled cell counts or cycle phase times for the cell population of interest in the conditions they are to be measured in. This may be based on published estimates, prior experiments on the same or closely related cell types, or pilot DPNL measurements performed using a conventional waiting time. Where there is a range of values, use the mean counts or times.
(2) Specify an optimization criterion that reflects the experimental objective. For example, one may seek to maximize the SNR associated with S-phase inference alone or adopt a composite objective that balances inference quality across multiple phases by assigning a weight to each. Metrics based solely on inferring an unknown total cell-cycle duration are not considered here in our fixed-period phase-focused approach, as they trivially favor the longest possible waiting times.
(3) Apply the optimization tool provided in the supporting material to find a candidate waiting time using the chosen relative weightings. The tool may be initialized either with labeled cell counts from a pilot experiment or with approximate phase-duration estimates, and it returns a recommended waiting time that optimizes the chosen criterion under the model assumptions based on our simulation data.
(4) Optionally, the protocol may be refined for further iterations using experimental data obtained with the recommended waiting time to update model inputs and reassess the optimal timing. In practice, a single iteration is likely to be sufficient.

Importantly, the optimization depends primarily on cohort transport through the cell-cycle phases relative to the pulse timings, and initial estimates should place the inference within a realistic parameter scale but do not need to be exact for the optimization to apply. Prior knowledge of the expected number of cells that can be collected and how many repeats can be performed is helpful for determining the exact optimal timing but is not essential.

The practical value of optimizing the waiting time $t_{\mathrm{wait}}$ can be understood by comparing the intrinsic variability of experimentally observed cell counts to the resolution of the parameter look-up grid. Table 1 reports mean EdU-single-positive cell count standard deviations obtained from an ensemble of simulated 3-repeat experiments for a representative parameter set, alongside the total variation in mean counts across the full parameter search space consistent with a 24-h cell cycle. For a waiting time that is too short, the standard deviation of the measured counts is comparable to—or exceeds—the variation in mean counts across the entire look-up grid (see Table 1), which is designed to encompass all reasonable cycle parameter combinations. In this regime, different parameter combinations become experimentally indistinguishable, limiting the effectiveness of any counts-based inference.

**Table 1:** Synthetic experiment outputs showing the impact of an optimal versus a non-optimal choice of $t_{\mathrm{wait}}$

$t_{\mathrm{wait}}$	$\sigma_{N\left({\text{EdU}}^{+}{\text{BrdU}}^{-}\right)}$	Grid range	SNR $\left(k_{S}\right)$
1 h	2.2	1.2	19.6
3 h	3.5	7.0	27.8

The standard deviation and search-grid range for the mean number of EdU^+^BrdU^−^ cells are shown, which illustrates how early times are limited by excessive noise compared to cell-count differences generated across a realistic parameter range. In the rightmost column, the SNR of parameter $k_{S}$ inference from Fig. 3 is shown. The tabulated data are average results over an ensemble of 3-repeat experimental trials using a 1-h (non-optimal) versus 3-h (optimal) time interval for an 11-h G_1_, 8-h S, and 5-h G_2_M phase system.

The limitations can in principle be partially mitigated by increasing the number of measured cells or the number of experimental repeats (see section SM7 for details); however, when these are constrained, the choice of $t_{\mathrm{wait}}$ becomes critical. By contrast, an optimized waiting time substantially increases the separation between mean responses across parameter space relative to experimental noise, improving identifiability even with limited data. Our optimization therefore identifies the best-case experimental configuration achievable under fixed sampling constraints. Further details on the suggested optimization protocol are available in section SM8.

## Conclusion

By modeling the cell cycle as a sequence of three transitions with noisy timing, we establish a proof of principle that the waiting time between labels in dual-pulse nucleoside labeling (DPNL) experiments can be nontrivially optimized to maximize the SNR of cell-cycle parameter inference. Using a minimal stochastic framework, we show that inference accuracy depends non-monotonically on pulse separation and that a well-defined optimal waiting time generically exists. Selecting this timing can improve S-phase inference by up to 50% relative to commonly used heuristic choices without increasing cell numbers or experimental repeats.

The simulation-based inference procedure used here is intentionally simple and closely mirrors the experimental mapping from labeled cell counts to kinetic parameters. As a consequence, it inherits the intrinsic identifiability limitations of DPNL data: information about the S phase dominates, while G_1_ and G_2_M parameters are only weakly constrained. These limitations are not specific to the inference method but arise from the restricted information content of population-level snapshot measurements. Within these bounds, our results demonstrate that experimental scheduling is a central and underutilized determinant of inference quality, often yielding larger gains than increasing inference complexity.

### Outlook

Although our analysis focuses on a minimal three-stage model and a specific implementation of DPNL, the underlying principle is general. Any labeling assay based on population-level snapshots is subject to a trade-off between cohort separation and the growth of stochastic variability, implying that optimal scheduling is a generic feature rather than a peculiarity of this system. The framework presented here provides a principled way to explore this trade-off using simple, system-specific models.

Several extensions can be pursued without altering this core philosophy. Incorporating additional experimental observables, such as nuclear DNA content measurements (27), would improve identifiability of individual phase durations while retaining the advantages of DPNL. More detailed cell-cycle models (18,19,28), including more tightly constrained phase durations or regulatory checkpoints, can be accommodated within the same input–output optimization framework provided that further experimental observables are available. Extending the approach to mixed or differentiating cell populations would further enable optimization of pulse scheduling for multiplexed experiments (29).

Taken together, these directions illustrate how simple stochastic modeling can inform experimental scheduling decisions under realistic constraints, offering a broadly applicable route to improving the precision of population-based labeling assays while remaining closely aligned with experimental practice.

## Data and code availability

Simulation data and all simulation, numeric, and plotting code used in this paper to generate data and Fig. 3 have been deposited in Zenodo: https://doi.org/10.5281/zenodo.19136034 and are publicly available as of this article’s publication date. Flow-cytometry data used in Fig. 1 *c* are available from the authors upon reasonable request.

## Acknowledgments

We have used the Imperial College Research Computing Service for this project (https://doi.org/10.14469/hpc/2232). For funding support, C.P., C.L.C., and C.F.L. thank CRUK Imperial Centre and NIHR Imperial BRC Data Science in Cancer Research Award 2020. C.P. acknowledges a CRUK Development grant, and C.L.C. acknowledges Wellcome Investigator award 212304/Z/18/Z and CRUK Programme Foundation award C36195/A26770.

## Author contributions

C.F.L. and C.L.C. designed the research; A.P. devised the approach and carried out the simulations, analytical calculations, and data analysis; A.P. and C.F.L. wrote the article; A.P. produced the figures and supporting material; C.P. and C.L.C. contributed expertise in the DPNL technique used to refine the approach; and C.P. collected the data and created the scatterplot in Fig. 1 *c*.

## Declaration of interests

The authors declare no competing interests.

## Supporting citations

References (30,31,32,33,34,35,36) appear in the supporting material.

## Supporting material

Document S1. Figures S1–S6 and supplemental materials and methods SM1–SM8

Document S2. Article plus supporting information

## References

[1] Ligasová A., Frydrych I., Koberna K. (2023). Basic Methods of Cell Cycle Analysis. Int. J. Mol. Sci..

[2] Bialic M., Al Ahmad Nachar B., Schwob E. (2022). Measuring S-Phase Duration from Asynchronous Cells Using Dual EdU-BrdU Pulse-Chase Labeling Flow Cytometry. Genes.

[3] Gitlin A.D., Shulman Z., Nussenzweig M.C. (2014). Clonal selection in the germinal center by regulated proliferation and hypermutation. Nature.

[4] Bannard O., McGowan S.J., Cyster J.G. (2016). Ubiquitin-mediated fluctuations in MHC class II facilitate efficient germinal center B cell responses. J. Exp. Med..

[5] Akinduro O., Weber T.S., Lo Celso C. (2018). Proliferation dynamics of acute myeloid leukaemia and haematopoietic progenitors competing for bone marrow space. Nat. Commun..

[6] Mickelson-Young L., Wear E., Thompson W. (2016). A flow cytometric method for estimating S-phase duration in plants. J. Exp. Bot..

[7] Wimber D.E., Quastler H. (1963). A 14C- and 3H-thymidine double labeling technique in the study of cell proliferation in Tradescantia root tips. Exp. Cell Res..

[8] Cappella P., Gasparri F., Moll J. (2008). A novel method based on click chemistry, which overcomes limitations of cell cycle analysis by classical determination of BrdU incorporation, allowing multiplex antibody staining. Cytometry. A..

[9] Kroll S., Char D., Kaleta-Michaels S. (1995). A stochastic model for dual label experiments: an analysis of the heterogeneity of S phase duration. Cell Prolif..

[10] Perrino G., Napolitano S., di Bernardo D. (2021). Automatic synchronisation of the cell cycle in budding yeast through closed-loop feedback control. Nat. Commun..

[11] Falcó C., Cohen D.J., Baker R. (2024). Quantifying cell cycle regulation by tissue crowding. Biophys. J..

[12] Martynoga B., Morrison H., Mason J.O. (2005). Foxg1 is required for specification of ventral telencephalon and region-specific regulation of dorsal telencephalic precursor proliferation and apoptosis. Dev. Biol..

[13] Somervaille T.C.P., Cleary M.L. (2006). Identification and characterization of leukemia stem cells in murine MLL-AF9 acute myeloid leukemia. Cancer Cell.

[14] Ma C., Gurkan-Cavusoglu E. (2024). A comprehensive review of computational cell cycle models in guiding cancer treatment strategies. npj Syst. Biol. Appl..

[15] Harris L., Zalucki O., Piper M. (2018). BrdU/EdU dual labeling to determine the cell-cycle dynamics of defined cellular subpopulations. J. Mol. Histol..

[16] Yates C.A., Ford M.J., Mort R.L. (2017). A Multi-stage Representation of Cell Proliferation as a Markov Process. Bull. Math. Biol..

[17] Araujo A.R., Gelens L., Santos S.D.M. (2016). Positive Feedback Keeps Duration of Mitosis Temporally Insulated from Upstream Cell-Cycle Events. Mol. Cell.

[18] Weber T.S., Jaehnert I., Carneiro J. (2014). Quantifying the Length and Variance of the Eukaryotic Cell Cycle Phases by a Stochastic Model and Dual Nucleoside Pulse Labelling. PLoS Comput. Biol..

[19] Jolly A., Fanti A.-K., Höfer T. (2022). CycleFlow simultaneously quantifies cell-cycle phase lengths and quiescence in vivo. Cell Rep. Methods.

[20] Weisel F.J., Zuccarino-Catania G.V., Shlomchik M.J. (2016). A Temporal Switch in the Germinal Center Determines Differential Output of Memory B and Plasma Cells. Immunity.

[21] Ritter M.A., Fowler J.F., Kinsella T.J. (1994). Tumor Cell Kinetics Using Two Labels and Flow Cytometry. Cytometry.

[22] Martí-Clúa J. (2023). Methods for Inferring Cell Cycle Parameters Using Thymidine Analogues. Biology.

[23] Greenberg A., Simon I. (2022). S Phase Duration Is Determined by Local Rate and Global Organization of Replication. Biology.

[24] Eastman A.E., Chen X., Guo S. (2020). Resolving Cell Cycle Speed in One Snapshot with a Live-Cell Fluorescent Reporter. Cell Rep..

[25] Reddy G.P., Tiarks C.Y., Quesenberry P.J. (1997). Cell cycle analysis and synchronization of pluripotent hematopoietic progenitor stem cells. Blood.

[26] Gillespie D.T. (2007). Stochastic Simulation of Chemical Kinetics. Annu. Rev. Phys. Chem..

[27] Roukos V., Pegoraro G., Misteli T. (2015). Cell cycle staging of individual cells by fluorescence microscopy. Nat. Protoc..

[28] Alsina A., Fumasoni M., Sartori P. (2025). Model-based inference of cell cycle dynamics captures alternations of the DNA replication programme. PLoS Comput. Biol..

[29] Rodríguez-Martínez M., Hills S.A., Svejstrup J.Q. (2020). Multiplex Cell Fate Tracking by Flow Cytometry. Methods Protoc..

[30] Reichenbach T., Mobilia M., Frey E. (2006). Coexistence versus extinction in the stochastic cyclic Lotka-Volterra model. Phys. Rev. E.

[31] Dobrinevski A., Frey E. (2012). Extinction in neutrally stable stochastic Lotka-Volterra models. Phys. Rev. E.

[32] Bernard S., Herzel H. (2009). Why Do Cells Cycle with a 24 Hour Period?. Genome Informatics.

[33] Malinin S.V., Chernyak V.Y. (2010). Transition times in the low-noise limit of stochastic dynamics. J. Chem. Phys..

[34] Schnoerr D., Sanguinetti G., Grima R. (2017). Approximation and inference methods for stochastic biochemical kinetics—a tutorial review. J. Phys. A: Math. Theor..

[35] Cooper G.M. (2000).

[36] Pant S. (2018). Information sensitivity functions to assess parameter information gain and identifiability of dynamical systems. J. R. Soc. Interface.

