# Supplementary material for: Optimizing scheduling in dual-pulse nucleoside labeling experiments for cell-cycle analysis
Source: Biophys J. 2026 Mar 27;125(9):2115–21. doi: 10.1016/j.bpj.2026.03.049 (PMC13351721; doi:10.1016/j.bpj.2026.03.049)
Supplement: Document S1. Figures S1–S6 and supplemental materials and methods SM1–SM8 [file mmc1.pdf]

Biophysical Journal, Volume *125*

Supplemental information

Optimizing scheduling in dual-pulse nucleoside labeling experiments for cell-cycle analysis

Alastar Phelan, Constandina Pospori, Cristina Lo Celso, and Chiu Fan Lee

Supporting Material:
Optimizing scheduling in dual-pulse nucleoside labeling experiments for cell cycle analysis

Alastar Phelan[1], Constandina Pospori[2], Cristina Lo Celso[2], Chiu Fan Lee[1]

[1]Department of Bioengineering, Imperial College London, South Kensington Campus, London SW7 2AZ, U.K.
[2]Department of Life Sciences, Imperial College London, South Kensington Campus, London SW7 2AZ, U.K.

SM1. STEADY GROWTH STATE

In contrast to a cyclic deterministic mass-conserving reaction scheme [1][2], the three stage cell cycle model has no steady state but instead can achieve balanced growth, which corresponds to the steady state of the normalized system

$$\mathbf{x}(t) = (x, y, z)^T(t) = \mathbf{N}(t)/N(t),$$

the number of cells in each phase of the cycle divided by the total number of cells at each time t.

With an exponential growth ansatz

$$\mathbf{N}(t) = N(0)e^{k_{G_2M}z(t)t}\mathbf{x}(t)$$

Differentiating with respect to time, the deterministic equations of motion from this ansatz become

$$\frac{d\mathbf{x}}{dt} + k_{G_2M}z\mathbf{x} + k_{G_2M}\dot{z}t\mathbf{x} = \begin{pmatrix} -k_{G_1} & 0 & 2k_{G_2M} \\ k_{G_1} & -k_S & 0 \\ 0 & k_S & -k_{G_2M} \end{pmatrix}\mathbf{x}$$

and the normalized system can be solved for a steady state $\dot{\mathbf{x}} = \mathbf{0}$ by $z \to \alpha_1$ where α_1 is the root of $-k_{G_1}k_S + (k_{G_1}k_S + k_{G_1}k_{G_2M} + k_Sk_{G_2M})\alpha + (k_{G_1} + k_S + k_{G_2M})k_{G_2M}\alpha^2 + k_{G_2M}^2\alpha^3 = 0$ located near $\alpha = 1$, and

$$x \to \frac{2\alpha_1 k_{G_2M}}{k_{G_1} + \alpha_1 k_{G_2M}}$$

$$y \to \frac{2\alpha_1 k_{G_1} k_{G_2M}}{(k_{G_1} + \alpha_1 k_{G_2M})(k_S + \alpha_1 k_{G_2M})}$$

For a 24 hour cell cycle period with G_1 lasting 11 hours, S 8 hours, G_2M 5 hours [3], meaning $k_{G_1} = 0.091$, $k_S = 0.13$, $k_{G_2M} = 0.20$, the renormalized steady state is situated at approximately $x \to 0.53$, $y \to 0.31$, $z \to 0.16$. Note that G_1 contains more cells than the other phases per hour of its duration. This is because the distribution of cells around the cycle at steady growth is biased towards earlier points in the cycle due to the generation of two new G_1 cells at the end of G_2M [4]. In the absence of any absorbing states since there is no cell death in our model, the stochastic trajectories remain close to the deterministic solution on average, within a thin tube of noise [5].

SM2. SOLVING THE FULL MASTER EQUATION

The following is intended to be self-contained, and contains technical detail that can be found in many pedagogical texts. For a more comprehensive reference on the topic, see [6], of which section 3 coincides with most of the technical detail of this section.

In terms of probability current, where state $\mathbf{N}$'s outgoing current via cycle phase advancement i is the product of the rate of exit via i given state $\mathbf{N}$ and the probability $P(\mathbf{N})$ to occupy that state: $J_i^{\downarrow}(\mathbf{N}, t) = \mathcal{W}_i(\mathbf{N})P(\mathbf{N}, t)$,

$$\frac{dP}{dt}(\mathbf{N}, t) = \sum_{i=1}^{3} J_i^{\uparrow}(\mathbf{N}, t) - J_i^{\downarrow}(\mathbf{N}, t) \tag{1}$$

$$J_i^{\downarrow}(\mathbf{N}, t) \equiv k_i N_i P(\mathbf{N}, t) \tag{2}$$

$$J_i^{\uparrow}(\mathbf{N}, t) \equiv \begin{cases} J_i^{\downarrow}(\mathbf{N} + \hat{\mathbf{x}}_i - \hat{\mathbf{x}}_{i+1}, t) & i = 1, 2 \\ J_3^{\downarrow}(\mathbf{N} + \hat{\mathbf{x}}_3 - 2\hat{\mathbf{x}}_1, t) & i = 3 \end{cases} \tag{3}$$

with $P(\mathbf{N}, t)$ the probability at time t to occupy a state described by vector $\mathbf{N} = (N_{G_1}, N_S, N_{G_2M})^T$, the total number of cells in each cycle phase; k_i the rate parameter for a cell advancing from phase i to $i+1$; $\hat{\mathbf{x}}_i$ represents a single cell in phase i. The expectation of a quantity at a time t can be solved by multiplying the master equation by that quantity and summing over states. For example, the mean number of G_1 phase cells at a time t can be calculated as

$$\sum_{N_{G_1}=0}^{\infty} \frac{dP}{dt}(\mathbf{N}, t) \cdot N_{G_1} = \sum_{N_{G_1}=0}^{\infty} N_{G_1} \sum_{i=1}^{3} J_i^{\uparrow}(\mathbf{N}, t) - J_i^{\downarrow}(\mathbf{N}, t)$$

Since the number of G_1 cells is just a quantity to be counted over, the time derivative and sum on the left-hand side can be commuted

$$\frac{d}{dt}\sum_{N_{G_1}=0}^{\infty} P(\mathbf{N}, t) \cdot N_{G_1} = \frac{d\langle N_{G_1}\rangle}{dt}$$

and expanding the probability current terms on the right hand side, with a change of index we find that the expression can be simplified

$$\sum_{N_{G_1}=0}^{\infty} N_{G_1} \sum_{i=1}^{3} k_i(N_i + 1)P(\mathbf{N} - \delta_i\mathbf{N}, t) - k_i N_i P(\mathbf{N}, t)$$

where $\delta_i\mathbf{N}$ represents the change of cell counts in each phase brought about by one cell making the phase advancement i as described in (3). The ith element of $\delta_i\mathbf{N}$ is always -1. Making the substitution $\mathbf{N} - \delta_i\mathbf{N} \to \mathbf{N}$, noting that boundary cases where $N_i = 0$ for any i naturally contribute zero rate due

$$\mathbf{K}_{full} = \begin{pmatrix} -k_{G_1} & 0 & 2k_{G_2M} & 0 & 0 & 0 & 0 & 0 & 0 \\ k_{G_1} & -k_S & 0 & 0 & 0 & 0 & 0 & 0 & 0 \\ 0 & k_S & -k_{G_2M} & 0 & 0 & 0 & 0 & 0 & 0 \\ k_{G_1} & 0 & 4k_{G_2M} & -2k_{G_1} & 0 & 0 & 0 & 0 & 4k_{G_2M} \\ -k_{G_1} & 0 & 0 & k_{G_1} & -k_{G_1}-k_S & 0 & 2k_{G_2M} & 0 & 0 \\ k_{G_1} & k_S & 0 & 0 & 2k_{G_1} & -2k_S & 0 & 0 & 0 \\ 0 & -k_S & 0 & 0 & 0 & k_S & -k_S-k_{G_2M} & 0 & k_{G_1} \\ 0 & k_S & k_{G_2M} & 0 & 0 & 0 & 2k_S & -2k_{G_2M} & 0 \\ 0 & 0 & -2k_{G_2M} & 0 & k_S & 0 & 0 & 2k_{G_2M} & -k_{G_1}-k_{G_2M} \end{pmatrix} \tag{4}$$

FIG. S1. Full rate matrix for the average dynamics of number of cells in each phase as well as their squares and cross-products, which are used to calculate the expected variance at a given time. The columns and rows proceed in the order $\{N_{G_1}, N_S, N_{G_2M}, N_{G_1}^2, N_{G_1}N_S, N_S^2, N_SN_{G_2M}, N_{G_2M}^2, N_{G_2M}N_{G_1}\}$

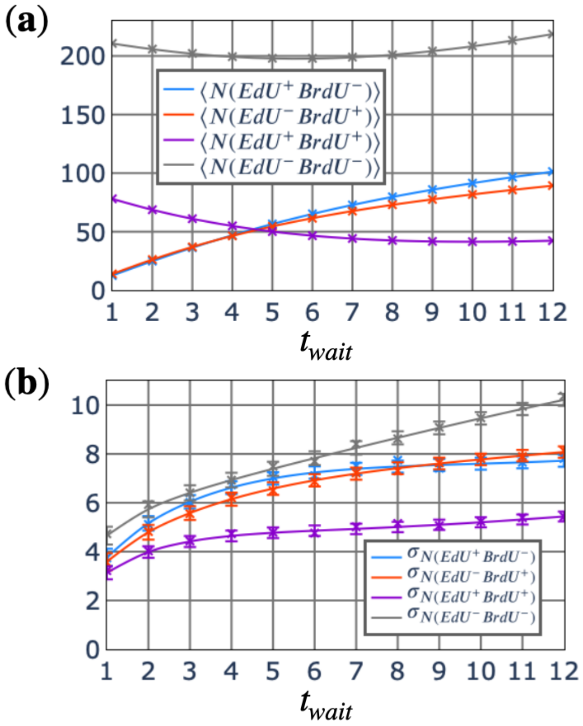

FIG. S2. **(a)** Average and **(b)** standard deviation of total cell counts across label combinations for different inter-pulse waiting times from simulation (crosses) and the analytic solution (lines). The counts are achieved by summing over phases for each of the four differently labeled cell populations. In hours, the series' phase times are $t_{G_1} = 11$, $t_S = 8$, $t_{G_2M} = 5$. Error bars are shown in plot (b) only, as deviations of up to 3% are seen in the standard deviation calculated from a sample of 10,000 simulations, while the average cell counts agree to within 0.1%.

to the mass action form of the master equation, the expression becomes

$$\sum_{N_{G_1}=0}^{\infty} \sum_{i=1}^{3} k_i N_i (N_{G_1} + \delta_i N_{G_1}) P(\mathbf{N}, t) - k_i N_i N_{G_1} P(\mathbf{N}, t)$$

now the sums can be evaluated and a cancellation made

$$= \sum_{N_{G_1}=0}^{\infty} \sum_{i=1}^{3} k_i N_i P(\mathbf{N}, t) \Big[(N_{G_1} + \delta_i N_{G_1}) - N_{G_1} \Big]$$

using that $\delta_1 N_{G_1} = -1$ and $\delta_3 N_{G_1} = +2$, we recover the deterministic noise-free limit of the model equations,

$$\frac{d\langle N_{G_1}\rangle}{dt} = 2k_3\langle N_{G_2M}\rangle - k_1\langle N_{G_1}\rangle \tag{5}$$

To calculate higher statistical moments, the same process is followed but with multiplying by $N_{G_1}^2$ for example instead. As a linear system, the moments of the 3-phase cell cycle model are summarized in equations (4) and (6) below.

$$\left[\frac{d}{dt} - \mathbf{K}_{full}\right] \begin{pmatrix} \langle N_{G_1}\rangle \\ \langle N_S\rangle \\ \langle N_{G_2M}\rangle \\ \langle N_{G_1}^2\rangle \\ \langle N_{G_1}N_S\rangle \\ \langle N_S^2\rangle \\ \langle N_SN_{G_2M}\rangle \\ \langle N_{G_2M}^2\rangle \\ \langle N_{G_2M}N_{G_1}\rangle \end{pmatrix} = \mathbf{0} \tag{6}$$

The resulting ODEs can be solved by a standard numerical integration technique for convenience, which show excellent agreement between Gillespie simulations and our analytic results from the approach outlined above, when quantifying the mean and standard deviation of the total number of cells with each label combination.

At the labeling times, the expected number of S phase cells $\langle N_S\rangle$ and expected squared number of S phase cells $\langle N_S^2\rangle$ is transferred to the population state of the newly labeled population, with these then set to zero in the source population for any labeling process i.e. colorless cells getting exposed to either label or EdU-positive cells exposed to BrdU in the late stages of an experiment, leaving behind zero cells in S phase. Cross-terms $\langle N_{G_1}N_S\rangle$ and $\langle N_SN_{G_2M}\rangle$ are set to zero. Summing over phases to produce the mean and standard deviation of the total number of

labeled cells gives the experimentally observable averaged cell counts at the end of a DPNL assay, plotted in (Fig. S2). The analytic approach recreates the average Gillespie simulation results closely, up to the noise remaining after 10^4-fold simulation.

Analytically proceeding from model parameters to the average and standard deviation of the labeled cells at the end of a DPNL experiment is limited to the forward direction for now, producing the ground truth look-up table used in this work. The subsequent Gillespie simulations generate a small number of outcomes as if running an experiment with 3 repeats, and the average cell counts from this noisy experiment are used to look up the most likely parameter values to have generated them from the ground truth table. A statistically identical ground truth table can be generated analytically or by bulk simulation as no approximation of the Master equation is needed for either method.

SM3. PARAMETER SWEEPS

Parameters (t_{G1}, t_S, t_{G2M}) were drawn from a hexagonal grid on the 2-simplex defined by $t_{G1}+t_S+t_{G2M}=24$h, centered on $(11.0, 8.0, 5.0)$ [7], resulting in 47, 35, and 13 unique values of each respective parameter, designed to cover a wide range of cell types with near 24 hour cycle periods. To convert to average rates (k_{G_1}, k_S, k_{G_2M}), the reciprocal of each time is taken. We analytically construct a dictionary of most likely cell counts at the end of an experiment with each model parameter set using the method in SM2.

Running 3 repeats thereafter as if running a real resource-limited experiment and selecting the closest entry in the dictionary to the average cell counts across those 3 repeats, the stochastic behavior will lead to deviations from the real model parameters being selected as most likely. The scoring function to work out the closest parameter combination is

$$\chi^2 = \sum_{label} \frac{(\bar{N}_{expt.} - \bar{N}_{true})^2}{\bar{N^2}_{true} - (\bar{N}_{true})^2}$$

where subscripts denote whether the number comes from the 3-repeat experiment (expt.) or the analytic look-up table (true). This process is repeated 1000 times per 3-repeat experiment per model parameter combination to find the mean and variance in inferred values of k_{G_1} and k_S. The ratio of the mean and standard deviation for each is then the inference signal-to-noise ratio for that model parameter.

SM4. STOCHASTIC SIMULATION ALGORITHM

The core Gillespie algorithm [8] for our simulated dual pulse nucleoside labeling experiments is as follows. We take a specified initial state (the 300-cell balanced growth state after rounding to the nearest cell, unless specified otherwise) and initial time $t = 0$. At each time until the end of the experiment,

1. Calculate the current propensities (bulk rates) of the system, $a_{ij} = k_i \cdot N_i^j$, where i iterates over the reaction index (equal to the source phase index), and j iterates over the four fluorescent label combinations i.e. cells which are positive for EdU, BrdU, both, or neither.
2. Draw a random number from an exponential distribution centered on $\left(\sum_{i,j} a_{ij}\right)^{-1}$, add it to the current time of the simulation.
3. Generate a random number from a uniform continuous distribution between 0 and $\sum_{i,j} a_{ij}$, find the first element in the cumulative sum which exceeds the random number in order to pick which single reaction takes place in this time interval, then add and take away the appropriate cells according to the stoichiometry of that reaction. For example, picking random number 2.1 with reaction propensities 1.1, 3.2 would result in the second possible reaction taking place.

Cells in the initial state of the system are exposed to EdU at time $t = 0$. This means making the assignments $N_S^{+-} = N_S^{--}$; $N_S^{--} = 0$. While the Gillespie population dynamics are running, at each time step, the following two conditional actions are performed to simulate the experimenter's labeling and harvesting steps.

- If the BrdU labeling time t_{wait} was just passed for the first time, set $N_S^{++} = N_S^{+-}$; $N_S^{-+} = N_S^{--}$; $N_S^{+-} = N_S^{--} = 0$.
- Otherwise, if the end time for the experiment, $t_{wait} + 0.5$ (cells cycle for a further half an hour for fixation as in real experiments), was just passed, then report the end state as the number of cells with each label combination: $\{N^j = \sum_i N_i^j\}\ \forall\ j$. End the simulation.
- Otherwise, go to next iteration.

SM5. INFERENCE ACCURACY

While the main focus of the paper is to optimize against noise in cell cycle kinetic parameter estimation from a DPNL experimental output, we have also verified that any bias in the average inferred parameters is acceptably low. The mean error of the inference reduces sharply with longer t_{wait} (Fig. S3), with the worst-case scenario close to just 10% error for G_1 phase duration inference for a waiting time of 1 hour. The worst-case scenario for S phase inference is a 2% error.

Intuitively, the longer the waiting time, the more growth in the cell population occurs, meaning relative fluctuations are suppressed and the system more closely resembles the analytically calculated average trajectories per repeat. The interaction of the dominant noise profile with the look-up grid width and

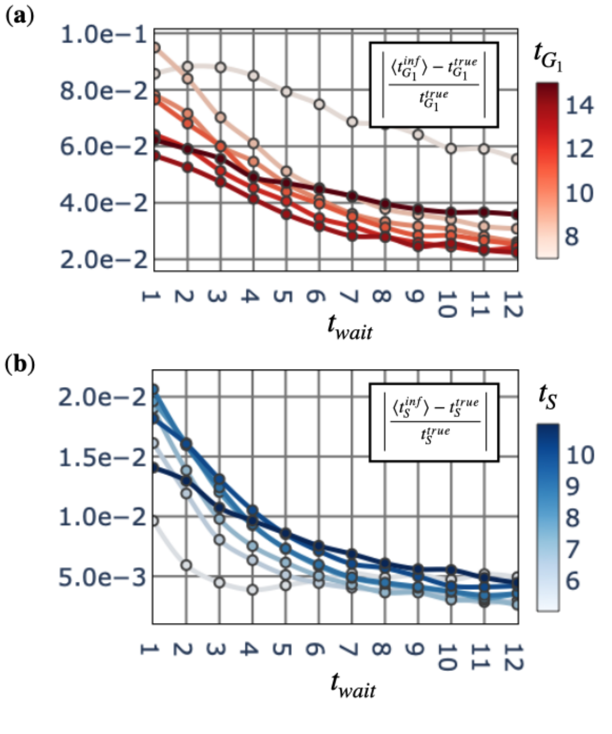

FIG. S3. *Squared mean error plots from parameter inference.* **(a)** Normalized average error squared in G_1 inference vs t_{wait}, binned into 7 G_1 duration groups. The edge of the parameter space with shortest G_1 shows**(b)** Normalized average error squared in S inference vs t_{wait}, binned into 7 S duration groups.

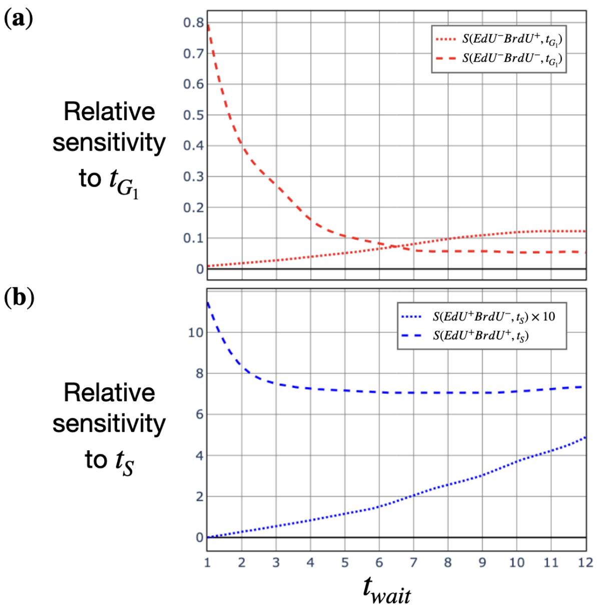

FIG. S4. Mean sensitivity of cell counts to changes of either **(a)** t_{G_1} or **(b)** t_S. Dotted lines show the mean sensitivity of single-positive cells to changes in the respective phase lengths, which is initially very small or even zero for a 1 hour t_{wait}, meaning small perturbations of phase times usually lead to no difference in the mean number of these cells. These curves monotonically increase with longer t_{wait}, while the sensitivity contribution by double-positive and double-negative cells begins at its highest due to the initially maximized population sizes. This tapers off to a steady value, where changes in noise levels are compensated by changes in counts. The scale of the curves of plot **b** is 5-10 times greater than **a**. The scale of the dotted line in plot **b** is magnified by 10 times for visibility.

spacing leads to moderate bias for short inter-pulse waiting times. It is particularly interesting that the monotonic increase in inference accuracy does not carry over to the Signal-to-Noise Ratio of the rate parameter inference - while biases shrink with increased population size, variability in the inferred rate parameter values have a peak or valley.

Sensitivity of labeled cell counts to parameters

The sensitivity of the labeled cell counts, precisely the change in labeled cell counts per change in parameter (t_{G_1} or t_S), normalized to the standard deviation of the cell count, measures the potential performance of an inference process on DPNL data.

$$S^{t_S}_{EdU^+BrdU^-} = \frac{\left(\frac{\partial \bar{N}(EdU^+BrdU^-)}{\partial t_S}\right)^2}{\sigma^2_{N(EdU^+BrdU^-)}} \tag{7}$$

The sensitivity of EdU^+BrdU^- counts to perturbations in S phase duration t_S is described in (7). Similar quantities are defined for all other label combinations and for G_1 phase perturbations. The most relevant sensitivities to the system are plotted in Fig. S4. Single-positive cells would not be present in a situation with a t_{wait} of zero hours, and their mean populations begin as very small at 1 hour t_{wait}, with high ($>100\%$) proportional noise due to influx from the initially large double-positive and double-negative populations, regardless of phase durations. The sensitivity of these cells to changes in phase times increases monotonically with larger t_{wait}, but sensitivity of double-positive and double-negative cells drops sharply at early times. The stage at which the combined sensitivity first reaches a high or low could explain the peak and trough behavior of Fig. 3 of the main text, but an exact mechanism for why longer t_{wait} results in poorer $SNR\{k_S\}$ past the peak involves the increasing degeneracy of the state space at late times and is beyond the scope of this work. It is likely that producing a statistic which measures the extent of cells' spread round the phases of the cycle to produce similar counts ratios across different parameter combinations, and the corresponding loss of original information from the EdU pulse in the final state of the system, is crucial to explaining this behavior.

The similar shapes of each corresponding curve between panels **(a)** and **(b)** in Fig. S4 imply that inference about G_1 in the DPNL setup is only possible due to information about S phase being linked to G_1 by the conservation of the total cycle time in our model, and the transfer of this information is weak as the scale of the sensitivity is 5-10 times smaller for G_1 compared to S phase.

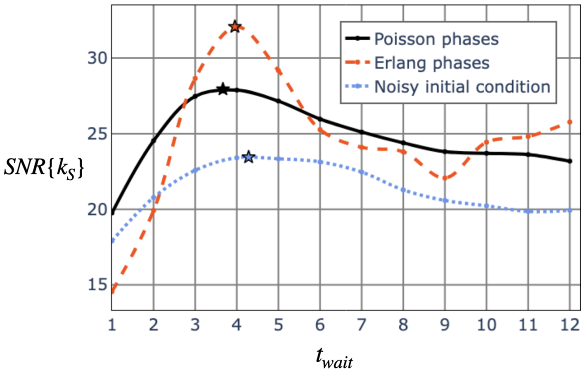

FIG. S5. Curves depicting a minor shift of k_S signal-to-noise ratio peak position across three models of the cell cycle. The base simulation dataset for the paper, with Poisson-distributed dwell times in each cell cycle phase, is marked in black. Using the same look-up method with new model-appropriate ground-truth data, we have tested two of the potential weaknesses of our original model as described in the methods section. Splitting each phase into 4 Poisson-distributed steps changes their overall dwell times to become Erlang-distributed (red, dashed line), with minimal probability mass near zero but maintaining a long tail. The SNR score of the peak is increased, with a narrower shape and minor shift to a slightly later time. The impact of noisy initial conditions (blue, dotted line) were tested by initializing with 20% of the noise of a 300-cell population grown from a single founding cell. This produced a broader, shorter peak, and weakly shifted the peak position to a later time. Data plotted use phase parameters within 5% of the mean values across the full dataset used in this work.

SM6. ROBUSTNESS TO DWELL-TIME DISTRIBUTIONS AND INITIAL-STATE UNCERTAINTY

The main text models progression through each cell cycle phase as a Poisson process, corresponding to exponentially distributed dwell times in each phase. While this assumption is standard and mathematically convenient at the population level, it implies a non-zero probability of arbitrarily short phase durations, which is biologically unrealistic at the individual level for controlled processes such as DNA replication or mitosis. In addition, simulations in the main text assume a deterministic initial phase distribution, whereas experimental cell populations—particularly those with small starting numbers—may exhibit substantial variability in their initial state.

To assess the sensitivity of our main conclusions to these modeling assumptions, we repeated the full simulation and inference pipeline using (i) a more realistic, non-exponential dwell-time model and (ii) stochastic initial conditions.

Erlang-distributed phase durations

To improve on the exponential dwell-time modeling choice while preserving computational tractability, we replaced each Poisson-distributed phase transition with an Erlang-distributed process. Specifically, each cell cycle phase was modeled as a sequence of four identical Poisson sub-steps, yielding an Erlang dwell-time distribution with minimal probability mass near zero duration and similar right skew to the exponential model, as commonly used in biophysical models of cell cycle progression [9]. Using this Erlang-based model, we analytically generated a new look-up table over waiting time and kinetic parameters, and repeated the grid-based inference on synthetic 3-repeat experiments exactly as in the main text.

Noisy initial conditions

To probe sensitivity to uncertainty in the initial phase distribution, we introduced stochasticity into the initial condition by sampling the starting phase counts with added noise corresponding to 20% of the variance expected if a 300-cell population were grown from a single founding cell, respecting the covariance of the cell counts per phase of the 3-stage cell cycle model. This represents a moderate and biologically plausible level of initial variability. New look-up tables were generated by repeat simulation under these noisy initial conditions, and inference was performed using the same protocol as in the main text.

Effect on optimal waiting-time inference

In both the Erlang-distributed and noisy-initial-condition cases, the detailed shape of the Signal-to-Noise Ratio (SNR) curves as a function of waiting time was altered relative to the baseline exponential phases model with a deterministic initial condition. However, the location of the S phase SNR maximum was relatively unchanged in the representative parameter combination subset (those within 5% of the mean values across the full dataset) (Fig. S5).

This demonstrates that while more realistic dwell-time distributions and initial-state variability can affect the magnitude and smoothness of the inferred SNR profiles, the central qualitative result of this work—the existence and location of a nontrivial optimal waiting time between labels—does not rely on the exponential phases approximation or deterministic initial conditions.

These results support the use of the simpler model in the main text as a proof-of-principle framework with lower computational burden, while indicating that the key optimization scenario is robust to biologically motivated extensions of the underlying cell cycle dynamics.

SM7. WEAK PEAK SHIFTS DUE TO NOISE SUPPRESSION

The magnitude of noise in measurements contributing to inference can tweak the optimal timing of a DPNL experiment. In DPNL, early times are dominated by the poor differentiability between cell counts produced by different parameter sets. Equivalently,

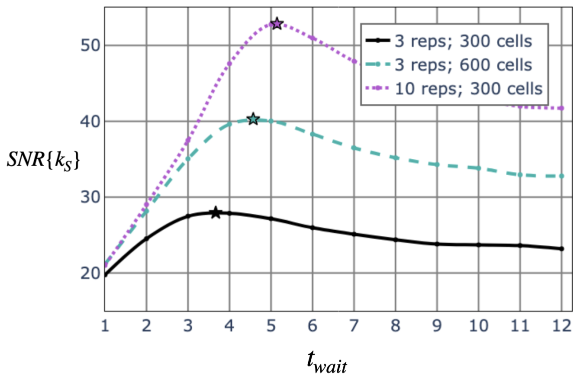

FIG. S6. Proportional noise in DPNL is decreased compared to the base setup (black solid curve) as described in the main text by drawing more repeat measurements (purple dotted curve) or collecting more cells (green dashed curve). This exposes more of the underlying identifiability structure of attempting to infer cell cycle rate parameters from mean labeled cell counts, leading to a peak time 1 hour later for a 24 hour cell cycle. Reducing noise weakly pushes optimal pulse timings later by raising the SNR ceiling of a system and allowing the parameter sensitivity to improve further before identifiability is diminished due to labeled cells redistributing diffusively among phases. This means that the resources available for an experiment can significantly affect choices about its optimization. Data plotted use phase parameters within 5% of the mean values across the full dataset used in this work.

the sensitivity in labeled cell counts to different model parameters is smaller than reasonable noise. With time, the separation of outcomes grows faster than the absolute noise in the system, and SNR improves. At late times, too much of the information from the initial EdU pulse is lost before the BrdU pulse is administered, with EdU-positive cells spreading around the cycle, leading to decreasing differences relative to noise in the numbers of labeled cells compared to shorter waiting times between pulses. We suggest that because decreasing noise in measurements by gathering more repeats or cells raises the maximum SNR achievable, it also means that identifiability loss due to diffusive spread of EdU-labeled cells around the cycle appears to happen later as there is further to fall (see Fig. S6) but an exact mechanism or correlation study is beyond the scope of this work. Sensitivity is maximized where measurable quantities vary most steeply while suffering lower noise [10], but a combined statistic for DPNL parameter sensitivity which recreates the $SNR\{k_2\}$ peak is nontrivial, requiring further investigation.

SM8. EXAMPLE IMPLEMENTATION OF WAITING-TIME OPTIMIZATION FOR DPNL EXPERIMENTS

To facilitate practical use of the optimization framework proposed in this work, we provide a simple, well-documented example script in Python with 11 example input cases using each of the available functions that recommends an optimal waiting time between nucleoside pulses based on pilot/prior Dual Pulse Nucleoside Labeling (DPNL) data. The script is intended to reflect a realistic experimental workflow, in which either an initial DPNL experiment is performed using a provisional waiting time, or published data on similar cells are used, and the resulting labeled cell counts or inferred phase times are used to guide the design of subsequent experiments.

The script takes as input data either:

- the waiting time used in the pilot experiment together with the observed counts (or proportions) of EdU single-positive, BrdU single-positive, and double-positive cells
- the estimated duration of G_1 and S phases along with the total cycle duration

These quantities are compared to a reference database of stochastic simulations generated under the cell cycle model described in the main text. By identifying simulations which produce similar relative labeling proportions or those which used similar phase timings relative to the total cycle period, the script recommends the waiting time that maximized the expected signal-to-noise ratio for inference of the S-phase duration (or a combined objective) in the closest-fit simulation data series. Since providing total cycle time allows for scaling the simulation optimum timing to suit this, it is recommended to use the phase times based method where possible. It is not in general possible to accurately estimate the duration of G_1 phase with only DPNL data, so if this information is used with the tool, it should come from another source such as single-cell timelapse imaging.

Importantly, the counts-based recommendation is based on a 24-hour cell cycle simulation. It may perform poorly for cells whose active cycle period is significantly different to this. The approach is applicable to a broad range of proliferating cell types, provided that the population is approximately homogeneous and continuously cycling while also being reasonably well contained (i.e., minimal cells are detracted non-uniformly from the gated counts by a change of location or phenotype in association with a phase completion). As a concrete example we include labeled cell count percentages from Fig. 1c, which were collected from a population of leukemic stem–like cells (LSCs) in mouse bone marrow (those with an Lin$^-$Sca-1$^+$c-Kit$^+$ phenotype in Acute Myeloid Leukemia). They represent a particularly good match to the model assumptions, as they are thought to proliferate continuously over experimental timescales and to exit the bone marrow compartment only rarely in the time before the bone marrow is annexed by leukemic cells [11].

We emphasize that this script is not intended to perform full parameter inference. Rather, it provides a lightweight, lookup-based tool that leverages pilot data to identify pulse timings that are expected to yield improved inference precision under realistic experimental constraints. The reference simulation data, optimized interval recommendation script, and example usage are available at this work's

https://github.com/atphelan/optimizing-dpnl-scheduling to enable straightforward adaptation to related experimental systems. A user-friendly version is available at https://atphelan.github.io/optimising-dpnl-scheduling-ui/.

---

[1] Tobias Reichenbach, Mauro Mobilia, and Erwin Frey. Coexistence versus extinction in the stochastic cyclic Lotka-Volterra model. Physical Review E, 74(5):051907, November 2006.

[2] Alexander Dobrinevski and Erwin Frey. Extiction in neutrally stable stochastic Lotka-Volterra models. Physical Review E, 85(5):051903, May 2012.

[3] Samuel Bernard and Hanspeter Herzel. Why Do Cells Cycle with a 24 Hour Period? Genome Informatics, 17(1):72–79, 2009.

[4] Anna Ligasová, Ivo Frydrych, and Karel Koberna. Basic Methods of Cell Cycle Analysis. International Journal of Molecular Sciences, 24(4):3674, February 2023.

[5] S. V. Malinin and V. Y. Chernyak. Transition times in the low-noise limit of stochastic dynamics. Journal of Chemical Physics, 132(1):014504, January 2010.

[6] David Schnoerr, Guido Sanguinetti, and Ramon Grima. Approximation and inference methods for stochastic biochemical kinetics—a tutorial review. Journal of Physics A: Mathematical and Theoretical, 50:093001, 2017.

[7] G. M. Cooper. The Cell: A Molecular Approach. Sunderland (MA): Sinauer Associates, 2 edition, 2000. Chapter: The Eukaryotic Cell Cycle.

[8] Daniel T. Gillespie. Stochastic Simulation of Chemical Kinetics. Annual Review Physical Chemistry, 58:35–55, October 2006.

[9] Adrien Jolly, Ann-Kathrin Fanti, Csilla Kongsaysak-Lengyel, Nina Claudino, Ines Gräßer, Nils B. Becker, and Thomas Höfer. CycleFlow simultaneously quantifies cell-cycle phase lengths and quiescence in vivo. Cell Reports Methods, 2(10):100315, October 2022.

[10] Sanjay Pant. Information sensitivity functions to assess parameter information gain and identifiability of dynamical systems. Journal of the Royal Society Interface, 15(142):20170871, May 2018.

[11] O. Akinduro, T. S. Weber, H. Ang, M. L. R. Haltalli, N. Ruivo, D. Duarte, N. M. Rashidi, E. D. Hawkins, K. R. Duffy, and C. Lo Celso. Proliferation dynamics of acute myeloid leukaemia and haematopoietic progenitors competing for bone marrow space. Nature Communications, 9(1):519, February 2018.
