# Supplementary material for: Optimizing scheduling in dual-pulse nucleoside labeling experiments for cell-cycle analysis
Source: Biophys J. 2026 Mar 27;125(9):2115–21. doi: 10.1016/j.bpj.2026.03.049 (PMC13351721; doi:10.1016/j.bpj.2026.03.049)
Supplement: Document S2. Article plus supporting information [file mmc2.pdf]

Biophysical Journal
Article

Biophysical Society

Optimizing scheduling in dual-pulse nucleoside labeling experiments for cell-cycle analysis

Alastar Phelan,[1] Constandina Pospori,[2,3] Cristina Lo Celso,[2,3] and Chiu Fan Lee[1,*]
[1]Department of Bioengineering, Imperial College London, London, UK; [2]Department of Life Sciences, Imperial College London, London, UK; and [3]The Francis Crick Institute, London, UK

ABSTRACT All eukaryotic cells go through a universal sequence of phases during their division cycle, where the phase timings vary according to cell type and state. Dual-pulse nucleoside labeling (DPNL) is a standard, widely applicable experimental DNA base-substituting technique to probe cell-cycle kinetics at the population level, including in living organisms. In such an experimental protocol, a key scheduling parameter is the choice of waiting time between the two labeling pulses. Here, we model population cell-cycle dynamics as a three-stage Poisson process with an idealized S-phase labeling step and use a simulation-based look-up procedure to demonstrate that the inter-pulse waiting time can be optimized to maximize the signal-to-noise ratio of inferred cycle parameters—an issue that is especially critical in DPNL experiments with limited cell numbers and replicates. An optimal choice of pulse scheduling typically improves S-phase time inference by 50% compared to a suboptimal choice. We further discuss the procedure to perform such a task in an experimentally relevant setting.

SIGNIFICANCE Dual-pulse nucleoside labeling (DPNL) is a widely used experimental technique for measuring cell-cycle dynamics, yet a key control parameter—the waiting time between labeling pulses—is typically chosen heuristically. Using a minimal stochastic model of cell-cycle progression, we show that inference accuracy depends non-monotonically on this waiting time and that an optimal pulse separation generically exists. Selecting this optimal timing can improve the signal-to-noise ratio of S-phase inference by up to 50% without increasing cell numbers or experimental repeats. Our results demonstrate that experimental scheduling is a critical and tunable component of quantitative inference in DPNL assays and provide a practical, model-guided framework for improving precision under experimentally realistic constraints.

INTRODUCTION

Tightly regulated cell divisions are crucial to the development and maintenance of all organisms. Before a cell can divide, it must go through multiple phases with carefully controlled checkpoints termed the G_1 (first gap phase), S (the synthesis phase when DNA is replicated), G_2 (second gap phase), and M (for mitosis). A quantitative understanding of how much time a cell spends in these distinct phases is fundamental to our understanding of basic cellular functions, from DNA replication to organelle duplication.

Given the importance of understanding cell-cycle dynamics, diverse experimental techniques have been developed to quantify the process (1) and among these the use of nucleoside substitution labeling is particularly popular due to its ease of use and high sensitivity (2). In such an experimental procedure, cells replicating their DNA in the S phase will incorporate modified nucleosides (e.g., 5-ethynyl-2′-deoxyuridine [EdU], a thymidine analog, Fig. 1 *a*) whose presence can then later be detected (e.g., via flow-cytometry methods). Detecting the amount of cells with these modified nucleosides in an EdU pulse-chase experiment has been a gold-standard approach to quantifying the duration they spend in the S phase (6).

To directly probe cell-cycle dynamics, a sequential substitution of nucleosides using two distinct labels (e.g., EdU and 5-bromo-2′-deoxyuridine [BrdU]), termed dual-pulse nucleoside labeling (DPNL), has been developed (7,8). In this experimental procedure, the second label is introduced into the system at a time t_{wait} after the introduction of the first label (Fig. 1 *b*). As a result, four distinctly labeled cell groups (Fig. 1 *c*) can be detected (as opposed to two in the single-labeling method), boosting the information output of the experiment and especially improving S-phase inference accuracy (9).

Submitted July 14, 2025, and accepted for publication March 23, 2026.
*Correspondence: c.lee@imperial.ac.uk
Editor: Guy Genin.
https://doi.org/10.1016/j.bpj.2026.03.049
Crown Copyright © 2026 Published by Elsevier Inc. on behalf of Biophysical Society.
This is an open access article under the CC BY license (http://creativecommons.org/licenses/by/4.0/).

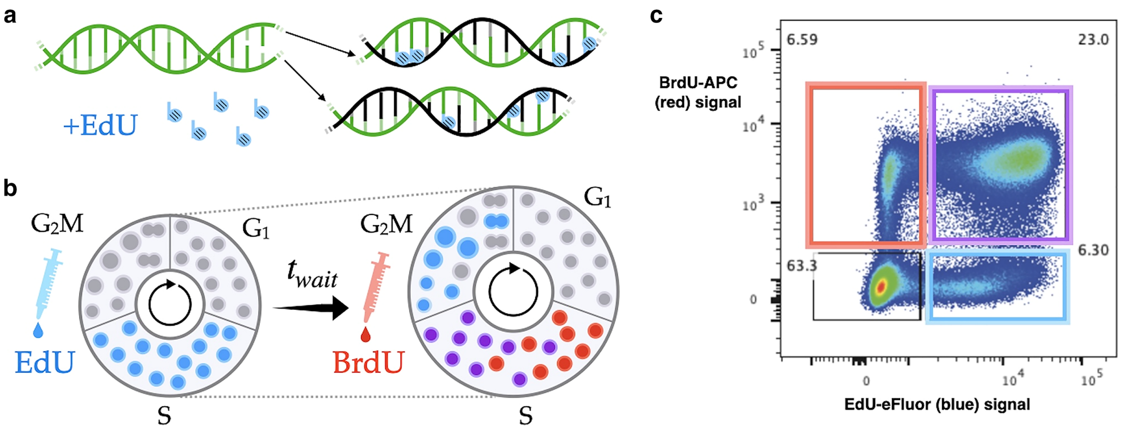

FIGURE 1 Nucleoside analog incorporation in dual-pulse nucleoside labeling (DPNL) to study the cell cycle. (a) If thymidine analog EdU (5-ethynyl-2′-deoxyuridine) is available during DNA synthesis, it is incorporated into each new half-strand produced. (b) In DPNL, the progress of cells through the cell cycle can be tracked by using a pulse of EdU (blue) followed by BrdU (5-bromo-2′-deoxyuridine, red) to show which cells have finished (blue), remained in (purple), or newly begun (red), DNA synthesis in the time between pulses. (c) The typical output of a dual-pulse labeling assay is a square-shaped distribution of fluorescence intensity, as measured by flow cytometry, which is proportional to the number of analog molecules. There are four density peaks at the coarsest level (3,4) corresponding to the colorings shown in (b). The percentage of cells with each fluorescence combination are shown in text near to each box. The data shown are from bone marrow-situated acute myeloid leukemia cells collected according to the method described in Akinduro et al. (5), with a 2-h waiting time.

Double-positive cells (purple, Fig. 1 *b* and *c*) are those which were in S phase during both EdU and BrdU exposure, and single-positive cells (blue, red in Fig. 1 *b* and *c*) are those which were only in S phase at either the EdU or the BrdU exposure time, meaning they were late S phase or early S phase, respectively, at the corresponding pulse time; otherwise, cells are double negative. For populations engaged in a synchronized cell cycle (10), only one of these four label states would be observed in a DPNL assay. While BrdU positivity at the end of a DPNL experiment implies that a cell is in S phase, both the EdU-single-positive and the double-negative populations are split between the G_2M and G_1 phases in continuously proliferating cell types. This output structure suggests that S-phase inference will be better constrained by DPNL data compared to G_1 and G_2M.

However, cells *in vivo* are rarely synchronized, and variability (even purely at the population level) in the times that cells within a population take to complete each phase, due to constraints such as tissue crowding (5,11) or mitogenic factors (12,13), remain a challenge for quantitative modeling where detailed timeseries data are scarce (14). Noisy cycle dynamics can have a large impact on inference about a whole population when the number of specific cells of interest (e.g., actively proliferating hematopoietic stem cells in a mouse) may be fewer than 1,000 cells (5). Compounding with this, the number of repeats of cells or tissues sampled is typically small in a given study, highlighting the need to optimize the accuracy of the experimental measurements. Indeed, a clear control parameter in this dual-labeling method is the waiting time t_{wait} between the introductions of the two labels. A longer t_{wait} allows more EdU-positive cells to leave S phase before the BrdU pulse, giving a smaller double-positive population, but larger single-positive populations, for t_{wait} shorter than S phase. However, how to choose t_{wait} to optimize the information output has thus far, to the best of our knowledge, not been thoroughly investigated. The typical constraints on t_{wait} are that it must be longer than the timescale for a pulse's nucleoside analogs to be incorporated into nascent DNA and shorter than the duration of S phase such that there is a double-labeled population from which to infer dynamic information. This can lead to timings from 1.5 to 3 h (15). Two hours is a common choice of t_{wait} due to the practicality of performing repeat experiments with this timing during a working day, without it being so short that single-labeled populations are too scarce to be reliable to infer dynamic parameters. Here, we perform this task and demonstrate, using simulation of a simple model of cell cycle (Fig. 2), how to find the t_{wait} that optimizes the signal-to-noise ratio (SNR) of the experimental measurements. Our work provides a proof of principle in how to use modeling to improve the precision of a widely used experimental method in the study of cell-cycle dynamics.

MATERIALS AND METHODS

Population-level cell-cycle progression model

We model the progress of the cell cycle through distinct phases equivalently to chemical reactions with rates k_i (Fig. 2), subject to fluctuations in the reaction kinetics. We have used a common simplification in the model, namely contracting G_2 and M phases into a single phase, termed G_2M. The two phases, usually the shortest in the cycle, are not readily distinguishable when looking solely at EdU fluorescence or DNA content (1,17), hence the phases are often modeled as one.

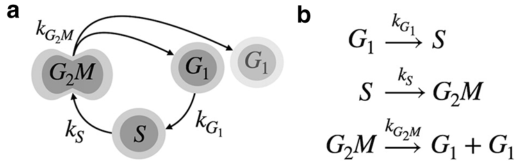

FIGURE 2 The three-species, three-parameter model of the cell cycle we have used in this work. (a) Cells in each stage i of the cycle advance to the next at a rate k_i, as a Poisson process. Two new cells are generated at the end of the cycle, both of which continue to advance through the cycle phases from the beginning, G_1 phase. (b) Reaction scheme describing the cell-cycle phase transitions. As a three-stage Poisson process, the total duration of the cycle is Erlang-distributed (16), while using the minimum number of parameters to keep results fully interpretable.

Cells are modeled as progressing through the cycle asynchronously, which is accepted for quantifying the cell cycle across a cell population in most cases (1,2,10). The model's strength is in its simplicity, where any other noisy cell-cycle model is necessarily more complex (18,19). The output of pure DPNL has two channels, giving four measurable quantities—the total number of cells with each fluorescence combination (Fig. 1 *b* and *c*)—meaning that any attempt to infer more than four independent model parameters from the output is likely to be difficult.

To find the model parameter-dependent statistics of the labeled cell counts at the end of DPNL experiments so that we can investigate how noise can be mitigated in the process, we model DPNL at the population level stochastically. The Master equation describing probabilistic trajectories of the population state, described by a vector $\mathbf{N} = (N_{G_1}, N_S, N_{G_2M})^T$, the number of cells in G_1, S and G_2M phase, respectively, is as follows:

$$\frac{dP}{dt}(\mathbf{N},t) = \sum_{i=1}^{3} \mathcal{W}_i(\mathbf{N} - \delta\mathbf{N}_i)P(\mathbf{N} - \delta\mathbf{N}_i, t) - \mathcal{W}_i(\mathbf{N})P(\mathbf{N},t) \quad (1)$$

where $P(\mathbf{N},t)$ is the probability at time t of having a population state $\mathbf{N}$. $\mathcal{W}_i(\mathbf{N}) = k_i N_i$ is the transition rate out of the state $\mathbf{N}$ via a single cell transition $\delta\mathbf{N}_i$, the loss of one cell and the gain of one or two in the next phase as described in the reaction scheme Fig. 2*b*, with $N_{i=1}$ corresponding to the number of G_1-phase cells, $k_{i=1}$ being their rate of progression to S phase, and so on for $i = 2,3$.

Idealized S-phase labeling during the cell cycle

For the labeling part of the model, we apply an EdU label to all cells in the S phase at time $t = 0$ only and apply BrdU to all cells in S phase and either EdU-positive or -negative at $t = t_{wait}$ only. In other words, labeling is assumed to be instantaneous and complete, as supported experimentally (5). We do not account for EdU dilution after cell division when analyzing flow-cytometry data, as the threshold for EdU positivity can be chosen while taking the dilution into account, with even the most diluted cells readily distinguished from EdU-negative cells. All labeled cells continue to advance through the cell cycle at unaltered rates until the end of the DPNL process, ultimately generating a separately cycling population for each label combination by this time.

Given the chemical master equations and labeling scheme above, we will now analytically calculate the results of an array of DPNL experiments.

Generating a look-up table from kinetic rates to DPNL outputs

As we focus here on population noise, which manifests itself most strongly in the study of small cell colonies, e.g., actively proliferating stem cells, we initialize our system with 300 cells, consistent with the reported numbers of hematopoietic stem cells entering S phase per hour in the mouse hind leg (5), as an example of a small population of replicating cells. When bulk measurements are made on more abundant cell types, the collected numbers of cells can reach tens of thousands, where the need for stringent optimization is smaller due to reduced relative population noise. The initial composition corresponds to the number of cells in each phase in the steady growth solution from solving for the dynamics of the deterministic model specified in Fig. 2 (see section SM1 of the supporting material for details). Further motivated by the typical experimental procedure (5,15,20), we have a 30-min interval after BrdU administration before counting up the cells with each label combination, which ensured the complete incorporation of BrdU in real experiments.

The total number of cells with each of the four label combinations—EdU single-positive (blue), BrdU single-positive (red), double-positive (purple), and double-negative (gray)—is the output of an experiment (see Fig. 1 *b* and *c*), which can be used to calculate individual phase durations, with the exact calculation depending on the cell type (7,15,18,21,22).

To probe the statistics of labeled cell outputs, we sweep across a range of scenarios by solving analytically (see section SM2 for details) across a grid of model parameters k_i and t_{wait}, while fixing the overall cell-cycle length to 24 h. This focuses the analysis on determining the lengths of each phase within a known total period, from which the results can be scaled proportionally to any other cycle length. The parameter sweep range considered (details in section SM3) is within the experimentally relevant range based on observed ratios between G_1-, S-, and G_2M-phase duration (Table S1 in Greenberg and Simon (23)) and keeping $t_{wait} \leq 12$ hours. Beyond this limit, a significant number of cells with a 24-h cycle could both exit S phase after receiving the EdU pulse and re-enter it in another lap of the cycle by the time of the BrdU pulse. Our analysis focuses on continuously proliferating cell populations, for which experimental protocols typically employ short interpulse intervals in order to mitigate this re-entry effect. In rapidly cycling systems, such as certain hematopoietic progenitor populations, the transit through G_2, M, and G_1 can in principle occur within a few hours, particularly under stimulatory conditions (24,25). Consequently, pulse separations on the order of 1–2 h are commonly adopted in practice to reduce the likelihood that cells labeled in the first pulse complete a full cycle and re-enter S phase before the second pulse. These cells could make cell-count outputs difficult to distinguish from a slower case where the same number of cells have remained in S phase throughout to register as double positive.

For each combination of model parameters, k_i and control parameter t_{wait}, we generate the mean and standard deviation of its output cell numbers' probability distributions through analytically solving the Master equation (Equation 1) using the standard method described in section SM2. This data set generated will serve as a ground-truth look-up table when we deal with typical experimental studies where the small number of repeats focuses our consideration on the high proportional noise in realistic measurements.

Inferring cell-cycle transition rates from typical experiments

We now use the ground-truth dataset generated as a dictionary to enable us to perform the inference of the model parameters k_i from the outputs of a typical experiment, which consists of only around 3–5 repeats. Hence, we perform 3-repeat simulations using a Gillespie algorithm (26) (see section SM4 for details) for each model parameter combination. For every 3-repeat trial, we look up the closest entry (see section SM3 for details) in the dictionary data previously collected to infer the most likely k_i. To obtain the statistics of this inference process, we perform a large number ($\sim 10^3$) of 3-repeat trials of simulations per model parameter combination to enable us to build up a distribution of estimated k_i from the simulated 3-repeat trials. Investigating these distributions will enable us to decipher the SNR of our inference process as a function of the waiting time t_{wait}.

RESULTS AND DISCUSSION

In a typical experiment, since the number of repeats is small, the inferred parameters k_i will deviate from the true cell-cycle rates due to intrinsic fluctuations.

Consider counting the number of cells in a population before and after a growth period, which is controlled by the experimenter, in order to infer the population's growth rate per cell. Waiting as long as possible is clearly the ideal case, as the expected deviation of the average division time of a cell is suppressed with longer growth times in line with the central limit theorem. However, in DPNL, the cell-cycle rate parameter inference cannot keep improving for longer waiting times because relatively fast-cycling EdU-positive cells reentering S phase for the BrdU pulse can be difficult to distinguish from slow-cycling cells that remained in S during that interval, increasing inference error and parameter sensitivity (5). Our simulation approach can quantify this trade-off.

To understand the impact of the stochasticity, we can use the inference SNR:

$$SNR\{k_i\} = \frac{k_i}{\sigma_{k_i}} \tag{2}$$

which corresponds to the ratio of the average most-likely k values and their standard deviations.

Here, we can quantify the SNRs accurately by comparing results from our 3-repeat *in silico* experiment with the known ground-truth rate parameters in our look-up.

In Fig. 3, we show the SNRs as a function of the waiting time t_{wait} for distinct set values of S-phase and G_1-phase periods, t_S (Fig. 3, main plot) and t_{G_1} (Fig. 3, inset), respectively. We find that *for any* set of initial ground-truth parameters (color bar in Fig. 3), $SNR\{k_S\}$ is generically non-monotonic with respect to t_{wait}, namely, there is indeed an optimal $SNR\{k_S\}$ at a nontrivial value of t_{wait}. Although $SNR\{k_{G_1}\}$ seems to show opposite trends to $SNR\{k_S\}$, the local minima in $SNR\{k_{G_1}\}$ do not coincide with the local maxima of $SNR\{k_S\}$, thus indicating that there exists generally an optimal t_{wait} that can optimize the SNR of the inference process, depending on the experimental focus (e.g., whether it is on t_S or on t_{G_1}). The peak position is robust to reasonable initial noise and more tightly controlled dwell times in each phase (see section SM6 for details).

We will now describe how such an optimization can be applied in a typical experiment, also shown in Fig. 4.

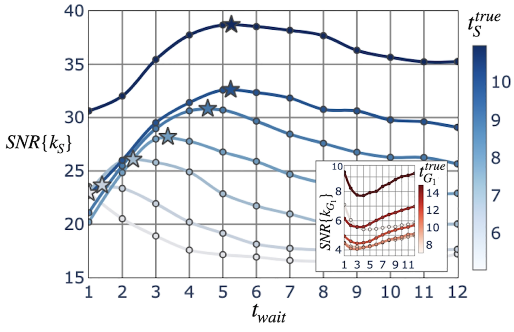

FIGURE 3 Signal-to-noise ratio (SNR) of the look-up inference process for model parameters k_i. Main panel: $SNR\{k_S\}$ versus t_{wait} (in hours) for different S-phase durations (color scale, in hours). A nontrivial S-phase-time-dependent optimum exists, reaching higher peak values for longer S-phase durations, with the optimal pulse interval simultaneously becoming longer. Inset: intermediate pulse interval times are unfavorable for G_1-phase duration inference across values of t_{G_1} (color scale, hours) and hint at an indirect trade-off with S-phase inference. The relative mean-squared inference errors for each parameter are shown in section SM5.

A protocol for optimizing the waiting time

(1) Obtain preliminary reference DPNL labeled cell counts or cycle phase times for the cell population of interest in the conditions they are to be measured in. This may be based on published estimates, prior experiments on the same or closely related cell types, or pilot DPNL measurements performed using a conventional waiting time. Where there is a range of values, use the mean counts or times.

(2) Specify an optimization criterion that reflects the experimental objective. For example, one may seek to maximize the SNR associated with S-phase inference alone or adopt a composite objective that balances inference quality across multiple phases by assigning a weight to each. Metrics based solely on inferring an unknown total cell-cycle duration are not considered here in our fixed-period phase-focused approach, as they trivially favor the longest possible waiting times.

(3) Apply the optimization tool provided in the supporting material to find a candidate waiting time using the

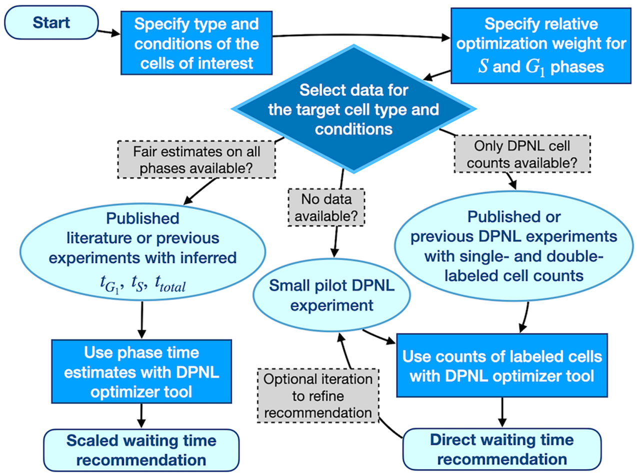

FIGURE 4 Workflow diagram illustrating optimization of cell-cycle measurement via our look-up-based tool. The relative optimization weights for S and G_1 phases should be specified when the optimizer tool is called, but prioritizing S phase is advised for a pure DPNL experiment with no supporting measurements that distinguish early and late cells within the cycle in order to separately count G_1- and G_2M-phase cells.

chosen relative weightings. The tool may be initialized either with labeled cell counts from a pilot experiment or with approximate phase-duration estimates, and it returns a recommended waiting time that optimizes the chosen criterion under the model assumptions based on our simulation data.

(4) Optionally, the protocol may be refined for further iterations using experimental data obtained with the recommended waiting time to update model inputs and reassess the optimal timing. In practice, a single iteration is likely to be sufficient.

Importantly, the optimization depends primarily on cohort transport through the cell-cycle phases relative to the pulse timings, and initial estimates should place the inference within a realistic parameter scale but do not need to be exact for the optimization to apply. Prior knowledge of the expected number of cells that can be collected and how many repeats can be performed is helpful for determining the exact optimal timing but is not essential.

The practical value of optimizing the waiting time t_{wait} can be understood by comparing the intrinsic variability of experimentally observed cell counts to the resolution of the parameter look-up grid. Table 1 reports mean EdU-single-positive cell count standard deviations obtained from an ensemble of simulated 3-repeat experiments for a representative parameter set, alongside the total variation in mean counts across the full parameter search space consistent with a 24-h cell cycle. For a waiting time that is too short, the standard deviation of the measured counts is comparable to—or exceeds—the variation in mean counts across the entire look-up grid (see Table 1), which is designed to encompass all reasonable cycle parameter combinations. In this regime, different parameter combinations become experimentally indistinguishable, limiting the effectiveness of any counts-based inference.

The limitations can in principle be partially mitigated by increasing the number of measured cells or the number of experimental repeats (see section SM7 for details); however, when these are constrained, the choice of t_{wait} becomes critical. By contrast, an optimized waiting time substantially increases the separation between mean responses across parameter space relative to experimental noise, improving identifiability even with limited data. Our optimization therefore identifies the best-case experimental configuration achievable under fixed sampling constraints. Further details on the suggested optimization protocol are available in section SM8.

TABLE 1 Synthetic experiment outputs showing the impact of an optimal versus a non-optimal choice of t_{wait}

t_{wait}	$\sigma_{N(\mathrm{EdU^+BrdU^-})}$	Grid range	SNR (k_S)
1 h	2.2	1.2	19.6
3 h	3.5	7.0	27.8

The standard deviation and search-grid range for the mean number of $\mathrm{EdU^+BrdU^-}$ cells are shown, which illustrates how early times are limited by excessive noise compared to cell-count differences generated across a realistic parameter range. In the rightmost column, the SNR of parameter k_S inference from Fig. 3 is shown. The tabulated data are average results over an ensemble of 3-repeat experimental trials using a 1-h (non-optimal) versus 3-h (optimal) time interval for an 11-h G_1, 8-h S, and 5-h G_2M phase system.

CONCLUSION

By modeling the cell cycle as a sequence of three transitions with noisy timing, we establish a proof of principle that the waiting time between labels in dual-pulse nucleoside labeling (DPNL) experiments can be nontrivially optimized to maximize the SNR of cell-cycle parameter inference. Using a minimal stochastic framework, we show that inference accuracy depends non-monotonically on pulse separation and that a well-defined optimal waiting time generically exists. Selecting this timing can improve S-phase inference by up to 50% relative to commonly used heuristic choices without increasing cell numbers or experimental repeats.

The simulation-based inference procedure used here is intentionally simple and closely mirrors the experimental mapping from labeled cell counts to kinetic parameters. As a consequence, it inherits the intrinsic identifiability limitations of DPNL data: information about the S phase dominates, while G_1 and G_2M parameters are only weakly constrained. These limitations are not specific to the inference method but arise from the restricted information content of population-level snapshot measurements. Within these bounds, our results demonstrate that experimental scheduling is a central and underutilized determinant of inference quality, often yielding larger gains than increasing inference complexity.

Outlook

Although our analysis focuses on a minimal three-stage model and a specific implementation of DPNL, the underlying principle is general. Any labeling assay based on population-level snapshots is subject to a trade-off between cohort separation and the growth of stochastic variability, implying that optimal scheduling is a generic feature rather than a peculiarity of this system. The framework presented here provides a principled way to explore this trade-off using simple, system-specific models.

Several extensions can be pursued without altering this core philosophy. Incorporating additional experimental observables, such as nuclear DNA content measurements (27), would improve identifiability of individual phase durations while retaining the advantages of DPNL. More detailed cell-cycle models (18,19,28), including more tightly constrained phase durations or regulatory checkpoints, can be accommodated within the same input–output optimization framework provided that further

experimental observables are available. Extending the approach to mixed or differentiating cell populations would further enable optimization of pulse scheduling for multiplexed experiments (29).

Taken together, these directions illustrate how simple stochastic modeling can inform experimental scheduling decisions under realistic constraints, offering a broadly applicable route to improving the precision of population-based labeling assays while remaining closely aligned with experimental practice.

DATA AND CODE AVAILABILITY

Simulation data and all simulation, numeric, and plotting code used in this paper to generate data and Fig. 3 have been deposited in Zenodo: https://doi.org/10.5281/zenodo.19136034 and are publicly available as of this article's publication date. Flow-cytometry data used in Fig. 1 *c* are available from the authors upon reasonable request.

ACKNOWLEDGMENTS

We have used the Imperial College Research Computing Service for this project (https://doi.org/10.14469/hpc/2232). For funding support, C.P., C.L.C., and C.F.L. thank CRUK Imperial Centre and NIHR Imperial BRC Data Science in Cancer Research Award 2020. C.P. acknowledges a CRUK Development grant, and C.L.C. acknowledges Wellcome Investigator award 212304/Z/18/Z and CRUK Programme Foundation award C36195/A26770.

AUTHOR CONTRIBUTIONS

C.F.L. and C.L.C. designed the research; A.P. devised the approach and carried out the simulations, analytical calculations, and data analysis; A.P. and C.F.L. wrote the article; A.P. produced the figures and supporting material; C.P. and C.L.C. contributed expertise in the DPNL technique used to refine the approach; and C.P. collected the data and created the scatterplot in Fig. 1 *c*.

DECLARATION OF INTERESTS

The authors declare no competing interests.

SUPPORTING MATERIAL

Supporting material can be found online at https://doi.org/10.1016/j.bpj.2026.03.049.

SUPPORTING CITATIONS

References (30–36) appear in the supporting material.

REFERENCES

1. Ligasová, A., I. Frydrych, and K. Koberna. 2023. Basic Methods of Cell Cycle Analysis. *Int. J. Mol. Sci.* 24:3674.
2. Bialic, M., B. Al Ahmad Nachar, …, E. Schwob. 2022. Measuring S-Phase Duration from Asynchronous Cells Using Dual EdU-BrdU Pulse-Chase Labeling Flow Cytometry. *Genes.* 13:408.
3. Gitlin, A. D., Z. Shulman, and M. C. Nussenzweig. 2014. Clonal selection in the germinal center by regulated proliferation and hypermutation. *Nature.* 509:637–640.
4. Bannard, O., S. J. McGowan, …, J. G. Cyster. 2016. Ubiquitin-mediated fluctuations in MHC class II facilitate efficient germinal center B cell responses. *J. Exp. Med.* 213:993–1009.
5. Akinduro, O., T. S. Weber, …, C. Lo Celso. 2018. Proliferation dynamics of acute myeloid leukaemia and haematopoietic progenitors competing for bone marrow space. *Nat. Commun.* 9:519.
6. Mickelson-Young, L., E. Wear, …, W. Thompson. 2016. A flow cytometric method for estimating S-phase duration in plants. *J. Exp. Bot.* 67:6077–6087.
7. Wimber, D. E., and H. Quastler. 1963. A 14C- and 3H-thymidine double labeling technique in the study of cell proliferation in Tradescantia root tips. *Exp. Cell Res.* 30:8–22.
8. Cappella, P., F. Gasparri, …, J. Moll. 2008. A novel method based on click chemistry, which overcomes limitations of cell cycle analysis by classical determination of BrdU incorporation, allowing multiplex antibody staining. *Cytometry. A.* 73:626–636.
9. Kroll, S., D. Char, and S. Kaleta-Michaels. 1995. A stochastic model for dual label experiments: an analysis of the heterogeneity of S phase duration. *Cell Prolif.* 28:545–567.
10. Perrino, G., S. Napolitano, …, D. di Bernardo. 2021. Automatic synchronisation of the cell cycle in budding yeast through closed-loop feedback control. *Nat. Commun.* 12:2452.
11. Falcó, C., D. J. Cohen, …, R. Baker. 2024. Quantifying cell cycle regulation by tissue crowding. *Biophys. J.* 123:1–10.
12. Martynoga, B., H. Morrison, …, J. O. Mason. 2005. Foxg1 is required for specification of ventral telencephalon and region-specific regulation of dorsal telencephalic precursor proliferation and apoptosis. *Dev. Biol.* 283:113–127.
13. Somervaille, T. C. P., and M. L. Cleary. 2006. Identification and characterization of leukemia stem cells in murine MLL-AF9 acute myeloid leukemia. *Cancer Cell.* 10:257–268.
14. Ma, C., and E. Gurkan-Cavusoglu. 2024. A comprehensive review of computational cell cycle models in guiding cancer treatment strategies. *npj Syst. Biol. Appl.* 10:71.
15. Harris, L., O. Zalucki, and M. Piper. 2018. BrdU/EdU dual labeling to determine the cell-cycle dynamics of defined cellular subpopulations. *J. Mol. Histol.* 49:229–234.
16. Yates, C. A., M. J. Ford, and R. L. Mort. 2017. A Multi-stage Representation of Cell Proliferation as a Markov Process. *Bull. Math. Biol.* 79:2905–2928.
17. Araujo, A. R., L. Gelens, …, S. D. M. Santos. 2016. Positive Feedback Keeps Duration of Mitosis Temporally Insulated from Upstream Cell-Cycle Events. *Mol. Cell.* 64:362–375.
18. Weber, T. S., I. Jaehnert, …, J. Carneiro. 2014. Quantifying the Length and Variance of the Eukaryotic Cell Cycle Phases by a Stochastic Model and Dual Nucleoside Pulse Labelling. *PLoS Comput. Biol.* 10:e1003616.
19. Jolly, A., A.-K. Fanti, …, T. Höfer. 2022. CycleFlow simultaneously quantifies cell-cycle phase lengths and quiescence in vivo. *Cell Rep. Methods.* 2:100315.
20. Weisel, F. J., G. V. Zuccarino-Catania, …, M. J. Shlomchik. 2016. A Temporal Switch in the Germinal Center Determines Differential Output of Memory B and Plasma Cells. *Immunity.* 44:116–130.
21. Ritter, M. A., J. F. Fowler, …, T. J. Kinsella. 1994. Tumor Cell Kinetics Using Two Labels and Flow Cytometry. *Cytometry.* 16:49–58.
22. Martí-Clúa, J. 2023. Methods for Inferring Cell Cycle Parameters Using Thymidine Analogues. *Biology.* 12:885.
23. Greenberg, A., and I. Simon. 2022. S Phase Duration Is Determined by Local Rate and Global Organization of Replication. *Biology.* 11:718.
24. Eastman, A. E., X. Chen, …, S. Guo. 2020. Resolving Cell Cycle Speed in One Snapshot with a Live-Cell Fluorescent Reporter. *Cell Rep.* 31:107804.

25. Reddy, G. P., C. Y. Tiarks, …, P. J. Quesenberry. 1997. Cell cycle analysis and synchronization of pluripotent hematopoietic progenitor stem cells. *Blood.* 90:2293–2299.

26. Gillespie, D. T. 2007. Stochastic Simulation of Chemical Kinetics. *Annu. Rev. Phys. Chem.* 58:35–55.

27. Roukos, V., G. Pegoraro, …, T. Misteli. 2015. Cell cycle staging of individual cells by fluorescence microscopy. *Nat. Protoc.* 10:334–348.

28. Alsina, A., M. Fumasoni, and P. Sartori. 2025. Model-based inference of cell cycle dynamics captures alternations of the DNA replication programme. *PLoS Comput. Biol.* 21:e1013570.

29. Rodríguez-Martínez, M., S. A. Hills, …, J. Q. Svejstrup. 2020. Multiplex Cell Fate Tracking by Flow Cytometry. *Methods Protoc.* 3:50.

30. Reichenbach, T., M. Mobilia, and E. Frey. 2006. Coexistence versus extinction in the stochastic cyclic Lotka-Volterra model. *Phys. Rev. E.* 74:051907.

31. Dobrinevski, A., and E. Frey. 2012. Extinction in neutrally stable stochastic Lotka-Volterra models. *Phys. Rev. E.* 85:051903.

32. Bernard, S., and H. Herzel. 2009. Why Do Cells Cycle with a 24 Hour Period? *Genome Informatics.* 17:72–79.

33. Malinin, S. V., and V. Y. Chernyak. 2010. Transition times in the low-noise limit of stochastic dynamics. *J. Chem. Phys.* 132:014504.

34. Schnoerr, D., G. Sanguinetti, and R. Grima. 2017. Approximation and inference methods for stochastic biochemical kinetics—a tutorial review. *J. Phys. A: Math. Theor.* 50:093001.

35. Cooper, G. M. 2000. The Cell: A Molecular Approach. Sinauer Associates.

36. Pant, S. 2018. Information sensitivity functions to assess parameter information gain and identifiability of dynamical systems. *J. R. Soc. Interface.* 15:20170871.

Biophysical Journal, Volume *125*

Supplemental information

Optimizing scheduling in dual-pulse nucleoside labeling experiments for cell-cycle analysis

Alastar Phelan, Constandina Pospori, Cristina Lo Celso, and Chiu Fan Lee

Supporting Material:

Optimizing scheduling in dual-pulse nucleoside labeling experiments for cell cycle analysis

Alastar Phelan[1], Constandina Pospori[2], Cristina Lo Celso[2], Chiu Fan Lee[1]

[1]Department of Bioengineering, Imperial College London, South Kensington Campus, London SW7 2AZ, U.K.
[2]Department of Life Sciences, Imperial College London, South Kensington Campus, London SW7 2AZ, U.K.

SM1. STEADY GROWTH STATE

In contrast to a cyclic deterministic mass-conserving reaction scheme [1][2], the three stage cell cycle model has no steady state but instead can achieve balanced growth, which corresponds to the steady state of the normalized system

$$\mathbf{x}(t) = (x, y, z)^T(t) = \mathbf{N}(t)/N(t),$$

the number of cells in each phase of the cycle divided by the total number of cells at each time t.

With an exponential growth ansatz

$$\mathbf{N}(t) = N(0)e^{k_{G_2M}z(t)t}\mathbf{x}(t)$$

Differentiating with respect to time, the deterministic equations of motion from this ansatz become

$$\frac{d\mathbf{x}}{dt} + k_{G_2M}z\mathbf{x} + k_{G_2M}\dot{z}t\mathbf{x} = \begin{pmatrix} -k_{G_1} & 0 & 2k_{G_2M} \\ k_{G_1} & -k_S & 0 \\ 0 & k_S & -k_{G_2M} \end{pmatrix}\mathbf{x}$$

and the normalized system can be solved for a steady state $\dot{\mathbf{x}} = \mathbf{0}$ by $z \to \alpha_1$ where α_1 is the root of $-k_{G_1}k_S + (k_{G_1}k_S + k_{G_1}k_{G_2M} + k_S k_{G_2M})\alpha + (k_{G_1} + k_S + k_{G_2M})k_{G_2M}\alpha^2 + k_{G_2M}^2\alpha^3 = 0$ located near $\alpha = 1$, and

$$x \to \frac{2\alpha_1 k_{G_2M}}{k_{G_1} + \alpha_1 k_{G_2M}}$$

$$y \to \frac{2\alpha_1 k_{G_1} k_{G_2M}}{(k_{G_1} + \alpha_1 k_{G_2M})(k_S + \alpha_1 k_{G_2M})}$$

For a 24 hour cell cycle period with G_1 lasting 11 hours, S 8 hours, G_2M 5 hours [3], meaning $k_{G_1} = 0.091$, $k_S = 0.13$, $k_{G_2M} = 0.20$, the renormalized steady state is situated at approximately $x \to 0.53$, $y \to 0.31$, $z \to 0.16$. Note that G_1 contains more cells than the other phases per hour of its duration. This is because the distribution of cells around the cycle at steady growth is biased towards earlier points in the cycle due to the generation of two new G_1 cells at the end of G_2M [4]. In the absence of any absorbing states since there is no cell death in our model, the stochastic trajectories remain close to the deterministic solution on average, within a thin tube of noise [5].

SM2. SOLVING THE FULL MASTER EQUATION

The following is intended to be self-contained, and contains technical detail that can be found in many pedagogical texts. For a more comprehensive reference on the topic, see [6], of which section 3 coincides with most of the technical detail of this section.

In terms of probability current, where state $\mathbf{N}$'s outgoing current via cycle phase advancement i is the product of the rate of exit via i given state $\mathbf{N}$ and the probability $P(\mathbf{N})$ to occupy that state: $J_i^{\downarrow}(\mathbf{N}, t) = \mathcal{W}_i(\mathbf{N})P(\mathbf{N}, t)$,

$$\frac{dP}{dt}(\mathbf{N}, t) = \sum_{i=1}^{3} J_i^{\uparrow}(\mathbf{N}, t) - J_i^{\downarrow}(\mathbf{N}, t) \quad (1)$$

$$J_i^{\downarrow}(\mathbf{N}, t) \equiv k_i N_i P(\mathbf{N}, t) \quad (2)$$

$$J_i^{\uparrow}(\mathbf{N}, t) \equiv \begin{cases} J_i^{\downarrow}(\mathbf{N} + \hat{\mathbf{x}}_i - \hat{\mathbf{x}}_{i+1}, t) & i = 1, 2 \\ J_3^{\downarrow}(\mathbf{N} + \hat{\mathbf{x}}_3 - 2\hat{\mathbf{x}}_1, t) & i = 3 \end{cases} \quad (3)$$

with $P(\mathbf{N}, t)$ the probability at time t to occupy a state described by vector $\mathbf{N} = (N_{G_1}, N_S, N_{G_2M})^T$, the total number of cells in each cycle phase; k_i the rate parameter for a cell advancing from phase i to $i+1$; $\hat{\mathbf{x}}_i$ represents a single cell in phase i. The expectation of a quantity at a time t can be solved by multiplying the master equation by that quantity and summing over states. For example, the mean number of G_1 phase cells at a time t can be calculated as

$$\sum_{N_{G_1}=0}^{\infty} \frac{dP}{dt}(\mathbf{N}, t) \cdot N_{G_1} = \sum_{N_{G_1}=0}^{\infty} N_{G_1} \sum_{i=1}^{3} J_i^{\uparrow}(\mathbf{N}, t) - J_i^{\downarrow}(\mathbf{N}, t)$$

Since the number of G_1 cells is just a quantity to be counted over, the time derivative and sum on the left-hand side can be commuted

$$\frac{d}{dt} \sum_{N_{G_1}=0}^{\infty} P(\mathbf{N}, t) \cdot N_{G_1} = \frac{d\langle N_{G_1} \rangle}{dt}$$

and expanding the probability current terms on the right hand side, with a change of index we find that the expression can be simplified

$$\sum_{N_{G_1}=0}^{\infty} N_{G_1} \sum_{i=1}^{3} k_i(N_i + 1)P(\mathbf{N} - \delta_i\mathbf{N}, t) - k_i N_i P(\mathbf{N}, t)$$

where $\delta_i\mathbf{N}$ represents the change of cell counts in each phase brought about by one cell making the phase advancement i as described in (3). The ith element of $\delta_i\mathbf{N}$ is always -1. Making the substitution $\mathbf{N} - \delta_i\mathbf{N} \to \mathbf{N}$, noting that boundary cases where $N_i = 0$ for any i naturally contribute zero rate due

$$\mathbf{K}_{full} = \begin{pmatrix} -k_{G_1} & 0 & 2k_{G_2M} & 0 & 0 & 0 & 0 & 0 & 0 \\ k_{G_1} & -k_S & 0 & 0 & 0 & 0 & 0 & 0 & 0 \\ 0 & k_S & -k_{G_2M} & 0 & 0 & 0 & 0 & 0 & 0 \\ k_{G_1} & 0 & 4k_{G_2M} & -2k_{G_1} & 0 & 0 & 0 & 0 & 4k_{G_2M} \\ -k_{G_1} & 0 & 0 & k_{G_1} & -k_{G_1}-k_S & 0 & 2k_{G_2M} & 0 & 0 \\ k_{G_1} & k_S & 0 & 0 & 2k_{G_1} & -2k_S & 0 & 0 & 0 \\ 0 & -k_S & 0 & 0 & 0 & k_S & -k_S-k_{G_2M} & 0 & k_{G_1} \\ 0 & k_S & k_{G_2M} & 0 & 0 & 0 & 2k_S & -2k_{G_2M} & 0 \\ 0 & 0 & -2k_{G_2M} & 0 & k_S & 0 & 0 & 2k_{G_2M} & -k_{G_1}-k_{G_2M} \end{pmatrix} \tag{4}$$

FIG. S1. Full rate matrix for the average dynamics of number of cells in each phase as well as their squares and cross-products, which are used to calculate the expected variance at a given time. The columns and rows proceed in the order $\{N_{G_1}, N_S, N_{G_2M}, N_{G_1}^2, N_{G_1}N_S, N_S^2, N_SN_{G_2M}, N_{G_2M}^2, N_{G_2M}N_{G_1}\}$

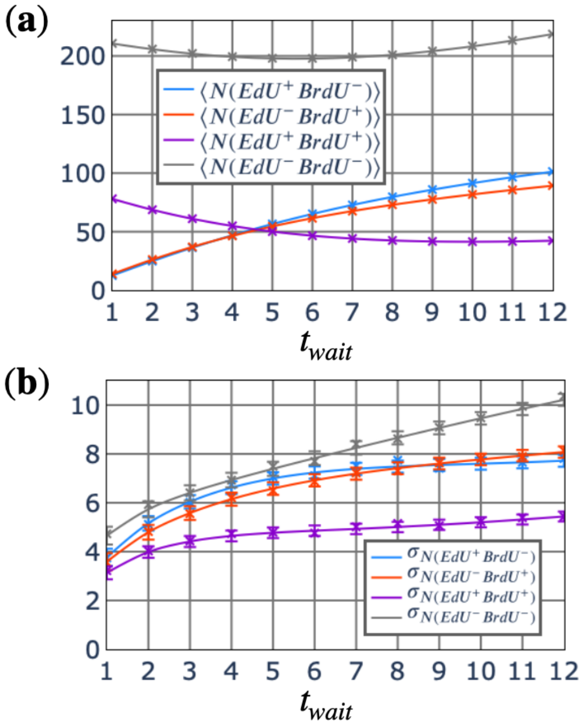

FIG. S2. **(a)** Average and **(b)** standard deviation of total cell counts across label combinations for different interpulse waiting times from simulation (crosses) and the analytic solution (lines). The counts are achieved by summing over phases for each of the four differently labeled cell populations. In hours, the series' phase times are $t_{G_1} = 11$, $t_S = 8$, $t_{G_2M} = 5$. Error bars are shown in plot (b) only, as deviations of up to 3% are seen in the standard deviation calculated from a sample of 10,000 simulations, while the average cell counts agree to within 0.1%.

to the mass action form of the master equation, the expression becomes

$$\sum_{N_{G_1}=0}^{\infty} \sum_{i=1}^{3} k_i N_i (N_{G_1} + \delta_i N_{G_1}) P(\mathbf{N}, t) - k_i N_i N_{G_1} P(\mathbf{N}, t)$$

now the sums can be evaluated and a cancellation made

$$= \sum_{N_{G_1}=0}^{\infty} \sum_{i=1}^{3} k_i N_i P(\mathbf{N}, t) \Big[(N_{G_1} + \delta_i N_{G_1}) - N_{G_1} \Big]$$

using that $\delta_1 N_{G_1} = -1$ and $\delta_3 N_{G_1} = +2$, we recover the deterministic noise-free limit of the model equations,

$$\frac{d\langle N_{G_1}\rangle}{dt} = 2k_3 \langle N_{G_2M}\rangle - k_1 \langle N_{G_1}\rangle \tag{5}$$

To calculate higher statistical moments, the same process is followed but with multiplying by $N_{G_1}^2$ for example instead. As a linear system, the moments of the 3-phase cell cycle model are summarized in equations (4) and (6) below.

$$\left[\frac{d}{dt} - \mathbf{K}_{full}\right] \begin{pmatrix} \langle N_{G_1}\rangle \\ \langle N_S\rangle \\ \langle N_{G_2M}\rangle \\ \langle N_{G_1}^2\rangle \\ \langle N_{G_1}N_S\rangle \\ \langle N_S^2\rangle \\ \langle N_S N_{G_2M}\rangle \\ \langle N_{G_2M}^2\rangle \\ \langle N_{G_2M}N_{G_1}\rangle \end{pmatrix} = \mathbf{0} \tag{6}$$

The resulting ODEs can be solved by a standard numerical integration technique for convenience, which show excellent agreement between Gillespie simulations and our analytic results from the approach outlined above, when quantifying the mean and standard deviation of the total number of cells with each label combination.

At the labeling times, the expected number of S phase cells $\langle N_S\rangle$ and expected squared number of S phase cells $\langle N_S^2\rangle$ is transferred to the population state of the newly labeled population, with these then set to zero in the source population for any labeling process i.e. colorless cells getting exposed to either label or EdU-positive cells exposed to BrdU in the late stages of an experiment, leaving behind zero cells in S phase. Cross-terms $\langle N_{G_1}N_S\rangle$ and $\langle N_S N_{G_2M}\rangle$ are set to zero. Summing over phases to produce the mean and standard deviation of the total number of

labeled cells gives the experimentally observable averaged cell counts at the end of a DPNL assay, plotted in (Fig. S2). The analytic approach recreates the average Gillespie simulation results closely, up to the noise remaining after 10^4-fold simulation.

Analytically proceeding from model parameters to the average and standard deviation of the labeled cells at the end of a DPNL experiment is limited to the forward direction for now, producing the ground truth look-up table used in this work. The subsequent Gillespie simulations generate a small number of outcomes as if running an experiment with 3 repeats, and the average cell counts from this noisy experiment are used to look up the most likely parameter values to have generated them from the ground truth table. A statistically identical ground truth table can be generated analytically or by bulk simulation as no approximation of the Master equation is needed for either method.

SM3. PARAMETER SWEEPS

Parameters (t_{G1}, t_S, t_{G2M}) were drawn from a hexagonal grid on the 2-simplex defined by $t_{G1} + t_S + t_{G2M} = 24$h, centered on $(11.0, 8.0, 5.0)$ [7], resulting in 47, 35, and 13 unique values of each respective parameter, designed to cover a wide range of cell types with near 24 hour cycle periods. To convert to average rates (k_{G_1}, k_S, k_{G_2M}), the reciprocal of each time is taken. We analytically construct a dictionary of most likely cell counts at the end of an experiment with each model parameter set using the method in SM2.

Running 3 repeats thereafter as if running a real resource-limited experiment and selecting the closest entry in the dictionary to the average cell counts across those 3 repeats, the stochastic behavior will lead to deviations from the real model parameters being selected as most likely. The scoring function to work out the closest parameter combination is

$$\chi^2 = \sum_{label} \frac{(\bar{N}_{expt.} - \bar{N}_{true})^2}{\bar{N^2}_{true} - (\bar{N}_{true})^2}$$

where subscripts denote whether the number comes from the 3-repeat experiment (expt.) or the analytic look-up table (true). This process is repeated 1000 times per 3-repeat experiment per model parameter combination to find the mean and variance in inferred values of k_{G_1} and k_S. The ratio of the mean and standard deviation for each is then the inference signal-to-noise ratio for that model parameter.

SM4. STOCHASTIC SIMULATION ALGORITHM

The core Gillespie algorithm [8] for our simulated dual pulse nucleoside labeling experiments is as follows. We take a specified initial state (the 300-cell balanced growth state after rounding to the nearest cell, unless specified otherwise) and initial time $t = 0$. At each time until the end of the experiment,

1. Calculate the current propensities (bulk rates) of the system, $a_{ij} = k_i \cdot N_i^j$, where i iterates over the reaction index (equal to the source phase index), and j iterates over the four fluorescent label combinations i.e. cells which are positive for EdU, BrdU, both, or neither.

2. Draw a random number from an exponential distribution centered on $\left(\sum_{i,j} a_{ij}\right)^{-1}$, add it to the current time of the simulation.

3. Generate a random number from a uniform continuous distribution between 0 and $\sum_{i,j} a_{ij}$, find the first element in the cumulative sum which exceeds the random number in order to pick which single reaction takes place in this time interval, then add and take away the appropriate cells according to the stoichiometry of that reaction. For example, picking random number 2.1 with reaction propensities 1.1, 3.2 would result in the second possible reaction taking place.

Cells in the initial state of the system are exposed to EdU at time $t = 0$. This means making the assignments $N_S^{+-} = N_S^{--}$; $N_S^{--} = 0$. While the Gillespie population dynamics are running, at each time step, the following two conditional actions are performed to simulate the experimenter's labeling and harvesting steps.

- If the BrdU labeling time t_{wait} was just passed for the first time, set $N_S^{++} = N_S^{+-}$; $N_S^{-+} = N_S^{--}$; $N_S^{+-} = N_S^{--} = 0$.
- Otherwise, if the end time for the experiment, $t_{wait} + 0.5$ (cells cycle for a further half an hour for fixation as in real experiments), was just passed, then report the end state as the number of cells with each label combination: $\{N^j = \sum_i N_i^j\} \; \forall \; j$. End the simulation.
- Otherwise, go to next iteration.

SM5. INFERENCE ACCURACY

While the main focus of the paper is to optimize against noise in cell cycle kinetic parameter estimation from a DPNL experimental output, we have also verified that any bias in the average inferred parameters is acceptably low. The mean error of the inference reduces sharply with longer t_{wait} (Fig. S3), with the worst-case scenario close to just 10% error for G_1 phase duration inference for a waiting time of 1 hour. The worst-case scenario for S phase inference is a 2% error.

Intuitively, the longer the waiting time, the more growth in the cell population occurs, meaning relative fluctuations are suppressed and the system more closely resembles the analytically calculated average trajectories per repeat. The interaction of the dominant noise profile with the look-up grid width and

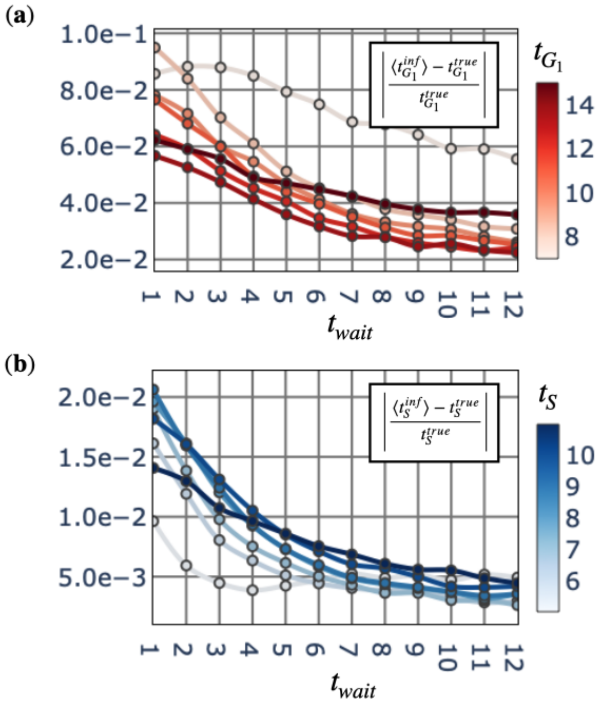

FIG. S3. *Squared mean error plots from parameter inference.* **(a)** Normalized average error squared in G_1 inference vs t_{wait}, binned into 7 G_1 duration groups. The edge of the parameter space with shortest G_1 shows**(b)** Normalized average error squared in S inference vs t_{wait}, binned into 7 S duration groups.

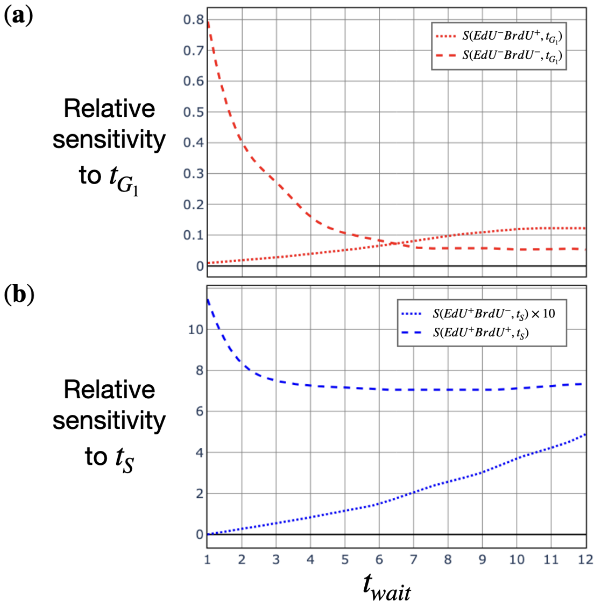

FIG. S4. Mean sensitivity of cell counts to changes of either **(a)** t_{G_1} or **(b)** t_S. Dotted lines show the mean sensitivity of single-positive cells to changes in the respective phase lengths, which is initially very small or even zero for a 1 hour t_{wait}, meaning small perturbations of phase times usually lead to no difference in the mean number of these cells. These curves monotonically increase with longer t_{wait}, while the sensitivity contribution by double-positive and double-negative cells begins at its highest due to the initially maximized population sizes. This tapers off to a steady value, where changes in noise levels are compensated by changes in counts. The scale of the curves of plot **b** is 5-10 times greater than **a**. The scale of the dotted line in plot **b** is magnified by 10 times for visibility.

spacing leads to moderate bias for short inter-pulse waiting times. It is particularly interesting that the monotonic increase in inference accuracy does not carry over to the Signal-to-Noise Ratio of the rate parameter inference - while biases shrink with increased population size, variability in the inferred rate parameter values have a peak or valley.

Sensitivity of labeled cell counts to parameters

The sensitivity of the labeled cell counts, precisely the change in labeled cell counts per change in parameter (t_{G_1} or t_S), normalized to the standard deviation of the cell count, measures the potential performance of an inference process on DPNL data.

$$S^{t_S}_{EdU^+BrdU^-} = \frac{\left(\frac{\partial \bar{N}(EdU^+BrdU^-)}{\partial t_S}\right)^2}{\sigma^2_{N(EdU^+BrdU^-)}} \qquad (7)$$

The sensitivity of EdU^+BrdU^- counts to perturbations in S phase duration t_S is described in (7). Similar quantities are defined for all other label combinations and for G_1 phase perturbations. The most relevant sensitivities to the system are plotted in Fig. S4. Single-positive cells would not be present in a situation with a t_{wait} of zero hours, and their mean populations begin as very small at 1 hour t_{wait}, with high ($> 100\%$) proportional noise due to influx from the initially large double-positive and double-negative populations, regardless of phase durations. The sensitivity of these cells to changes in phase times increases monotonically with larger t_{wait}, but sensitivity of double-positive and double-negative cells drops sharply at early times. The stage at which the combined sensitivity first reaches a high or low could explain the peak and trough behavior of Fig. 3 of the main text, but an exact mechanism for why longer t_{wait} results in poorer $SNR\{k_S\}$ past the peak involves the increasing degeneracy of the state space at late times and is beyond the scope of this work. It is likely that producing a statistic which measures the extent of cells' spread round the phases of the cycle to produce similar counts ratios across different parameter combinations, and the corresponding loss of original information from the EdU pulse in the final state of the system, is crucial to explaining this behavior.

The similar shapes of each corresponding curve between panels **(a)** and **(b)** in Fig. S4 imply that inference about G_1 in the DPNL setup is only possible due to information about S phase being linked to G_1 by the conservation of the total cycle time in our model, and the transfer of this information is weak as the scale of the sensitivity is 5-10 times smaller for G_1 compared to S phase.

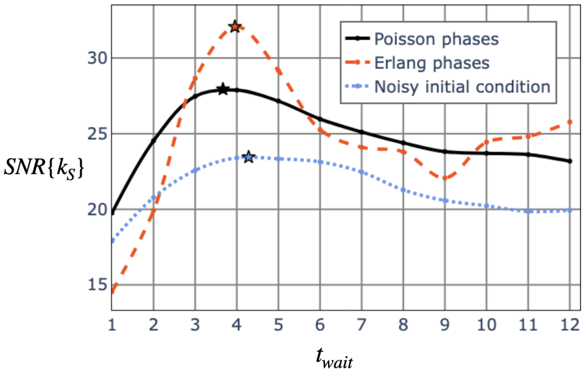

FIG. S5. Curves depicting a minor shift of k_S signal-to-noise ratio peak position across three models of the cell cycle. The base simulation dataset for the paper, with Poisson-distributed dwell times in each cell cycle phase, is marked in black. Using the same look-up method with new model-appropriate ground-truth data, we have tested two of the potential weaknesses of our original model as described in the methods section. Splitting each phase into 4 Poisson-distributed steps changes their overall dwell times to become Erlang-distributed (red, dashed line), with minimal probability mass near zero but maintaining a long tail. The SNR score of the peak is increased, with a narrower shape and minor shift to a slightly later time. The impact of noisy initial conditions (blue, dotted line) were tested by initializing with 20% of the noise of a 300-cell population grown from a single founding cell. This produced a broader, shorter peak, and weakly shifted the peak position to a later time. Data plotted use phase parameters within 5% of the mean values across the full dataset used in this work.

SM6. ROBUSTNESS TO DWELL-TIME DISTRIBUTIONS AND INITIAL-STATE UNCERTAINTY

The main text models progression through each cell cycle phase as a Poisson process, corresponding to exponentially distributed dwell times in each phase. While this assumption is standard and mathematically convenient at the population level, it implies a non-zero probability of arbitrarily short phase durations, which is biologically unrealistic at the individual level for controlled processes such as DNA replication or mitosis. In addition, simulations in the main text assume a deterministic initial phase distribution, whereas experimental cell populations—particularly those with small starting numbers—may exhibit substantial variability in their initial state.

To assess the sensitivity of our main conclusions to these modeling assumptions, we repeated the full simulation and inference pipeline using (i) a more realistic, non-exponential dwell-time model and (ii) stochastic initial conditions.

Erlang-distributed phase durations

To improve on the exponential dwell-time modeling choice while preserving computational tractability, we replaced each Poisson-distributed phase transition with an Erlang-distributed process. Specifically, each cell cycle phase was modeled as a sequence of four identical Poisson sub-steps, yielding an Erlang dwell-time distribution with minimal probability mass near zero duration and similar right skew to the exponential model, as commonly used in biophysical models of cell cycle progression [9]. Using this Erlang-based model, we analytically generated a new look-up table over waiting time and kinetic parameters, and repeated the grid-based inference on synthetic 3-repeat experiments exactly as in the main text.

Noisy initial conditions

To probe sensitivity to uncertainty in the initial phase distribution, we introduced stochasticity into the initial condition by sampling the starting phase counts with added noise corresponding to 20% of the variance expected if a 300-cell population were grown from a single founding cell, respecting the covariance of the cell counts per phase of the 3-stage cell cycle model. This represents a moderate and biologically plausible level of initial variability. New look-up tables were generated by repeat simulation under these noisy initial conditions, and inference was performed using the same protocol as in the main text.

Effect on optimal waiting-time inference

In both the Erlang-distributed and noisy-initial-condition cases, the detailed shape of the Signal-to-Noise Ratio (SNR) curves as a function of waiting time was altered relative to the baseline exponential phases model with a deterministic initial condition. However, the location of the S phase SNR maximum was relatively unchanged in the representative parameter combination subset (those within 5% of the mean values across the full dataset) (Fig. S5).

This demonstrates that while more realistic dwell-time distributions and initial-state variability can affect the magnitude and smoothness of the inferred SNR profiles, the central qualitative result of this work—the existence and location of a nontrivial optimal waiting time between labels—does not rely on the exponential phases approximation or deterministic initial conditions.

These results support the use of the simpler model in the main text as a proof-of-principle framework with lower computational burden, while indicating that the key optimization scenario is robust to biologically motivated extensions of the underlying cell cycle dynamics.

SM7. WEAK PEAK SHIFTS DUE TO NOISE SUPPRESSION

The magnitude of noise in measurements contributing to inference can tweak the optimal timing of a DPNL experiment. In DPNL, early times are dominated by the poor differentiability between cell counts produced by different parameter sets. Equivalently,

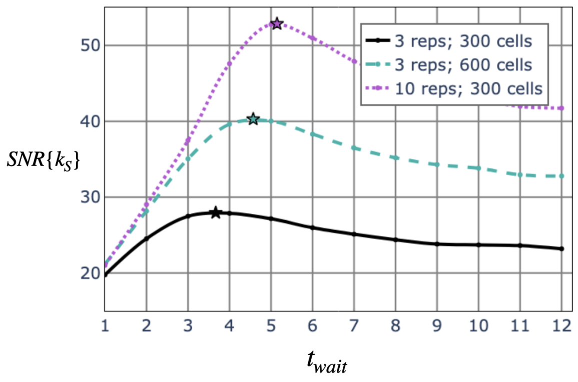

FIG. S6. Proportional noise in DPNL is decreased compared to the base setup (black solid curve) as described in the main text by drawing more repeat measurements (purple dotted curve) or collecting more cells (green dashed curve). This exposes more of the underlying identifiability structure of attempting to infer cell cycle rate parameters from mean labeled cell counts, leading to a peak time 1 hour later for a 24 hour cell cycle. Reducing noise weakly pushes optimal pulse timings later by raising the SNR ceiling of a system and allowing the parameter sensitivity to improve further before identifiability is diminished due to labeled cells redistributing diffusively among phases. This means that the resources available for an experiment can significantly affect choices about its optimization. Data plotted use phase parameters within 5% of the mean values across the full dataset used in this work.

the sensitivity in labeled cell counts to different model parameters is smaller than reasonable noise. With time, the separation of outcomes grows faster than the absolute noise in the system, and SNR improves. At late times, too much of the information from the initial EdU pulse is lost before the BrdU pulse is administered, with EdU-positive cells spreading around the cycle, leading to decreasing differences relative to noise in the numbers of labeled cells compared to shorter waiting times between pulses. We suggest that because decreasing noise in measurements by gathering more repeats or cells raises the maximum SNR achievable, it also means that identifiability loss due to diffusive spread of EdU-labeled cells around the cycle appears to happen later as there is further to fall (see Fig. S6) but an exact mechanism or correlation study is beyond the scope of this work. Sensitivity is maximized where measurable quantities vary most steeply while suffering lower noise [10], but a combined statistic for DPNL parameter sensitivity which recreates the $SNR\{k_2\}$ peak is nontrivial, requiring further investigation.

SM8. EXAMPLE IMPLEMENTATION OF WAITING-TIME OPTIMIZATION FOR DPNL EXPERIMENTS

To facilitate practical use of the optimization framework proposed in this work, we provide a simple, well-documented example script in Python with 11 example input cases using each of the available functions that recommends an optimal waiting time between nucleoside pulses based on pilot/prior Dual Pulse Nucleoside Labeling (DPNL) data. The script is intended to reflect a realistic experimental workflow, in which either an initial DPNL experiment is performed using a provisional waiting time, or published data on similar cells are used, and the resulting labeled cell counts or inferred phase times are used to guide the design of subsequent experiments.

The script takes as input data either:

- the waiting time used in the pilot experiment together with the observed counts (or proportions) of EdU single-positive, BrdU single-positive, and double-positive cells
- the estimated duration of G_1 and S phases along with the total cycle duration

These quantities are compared to a reference database of stochastic simulations generated under the cell cycle model described in the main text. By identifying simulations which produce similar relative labeling proportions or those which used similar phase timings relative to the total cycle period, the script recommends the waiting time that maximized the expected signal-to-noise ratio for inference of the S-phase duration (or a combined objective) in the closest-fit simulation data series. Since providing total cycle time allows for scaling the simulation optimum timing to suit this, it is recommended to use the phase times based method where possible. It is not in general possible to accurately estimate the duration of G_1 phase with only DPNL data, so if this information is used with the tool, it should come from another source such as single-cell timelapse imaging.

Importantly, the counts-based recommendation is based on a 24-hour cell cycle simulation. It may perform poorly for cells whose active cycle period is significantly different to this. The approach is applicable to a broad range of proliferating cell types, provided that the population is approximately homogeneous and continuously cycling while also being reasonably well contained (i.e., minimal cells are detracted non-uniformly from the gated counts by a change of location or phenotype in association with a phase completion). As a concrete example we include labeled cell count percentages from Fig. 1c, which were collected from a population of leukemic stem–like cells (LSCs) in mouse bone marrow (those with an Lin$^-$Sca-1$^+$c-Kit$^+$ phenotype in Acute Myeloid Leukemia). They represent a particularly good match to the model assumptions, as they are thought to proliferate continuously over experimental timescales and to exit the bone marrow compartment only rarely in the time before the bone marrow is annexed by leukemic cells [11].

We emphasize that this script is not intended to perform full parameter inference. Rather, it provides a lightweight, lookup-based tool that leverages pilot data to identify pulse timings that are expected to yield improved inference precision under realistic experimental constraints. The reference simulation data, optimized interval recommendation script, and example usage are available at this work's

https://github.com/atphelan/optimizing-dpnl-scheduling to enable straightforward adaptation to related experimental systems. A user-friendly version is available at https://atphelan.github.io/optimising-dpnl-scheduling-ui/.

---

[1] Tobias Reichenbach, Mauro Mobilia, and Erwin Frey. Coexistence versus extinction in the stochastic cyclic Lotka-Volterra model. Physical Review E, 74(5):051907, November 2006.

[2] Alexander Dobrinevski and Erwin Frey. Extiction in neutrally stable stochastic Lotka-Volterra models. Physical Review E, 85(5):051903, May 2012.

[3] Samuel Bernard and Hanspeter Herzel. Why Do Cells Cycle with a 24 Hour Period? Genome Informatics, 17(1):72–79, 2009.

[4] Anna Ligasová, Ivo Frydrych, and Karel Koberna. Basic Methods of Cell Cycle Analysis. International Journal of Molecular Sciences, 24(4):3674, February 2023.

[5] S. V. Malinin and V. Y. Chernyak. Transition times in the low-noise limit of stochastic dynamics. Journal of Chemical Physics, 132(1):014504, January 2010.

[6] David Schnoerr, Guido Sanguinetti, and Ramon Grima. Approximation and inference methods for stochastic biochemical kinetics—a tutorial review. Journal of Physics A: Mathematical and Theoretical, 50:093001, 2017.

[7] G. M. Cooper. The Cell: A Molecular Approach. Sunderland (MA): Sinauer Associates, 2 edition, 2000. Chapter: The Eukaryotic Cell Cycle.

[8] Daniel T. Gillespie. Stochastic Simulation of Chemical Kinetics. Annual Review Physical Chemistry, 58:35–55, October 2006.

[9] Adrien Jolly, Ann-Kathrin Fanti, Csilla Kongsaysak-Lengyel, Nina Claudino, Ines Gräßer, Nils B. Becker, and Thomas Höfer. CycleFlow simultaneously quantifies cell-cycle phase lengths and quiescence in vivo. Cell Reports Methods, 2(10):100315, October 2022.

[10] Sanjay Pant. Information sensitivity functions to assess parameter information gain and identifiability of dynamical systems. Journal of the Royal Society Interface, 15(142):20170871, May 2018.

[11] O. Akinduro, T. S. Weber, H. Ang, M. L. R. Haltalli, N. Ruivo, D. Duarte, N. M. Rashidi, E. D. Hawkins, K. R. Duffy, and C. Lo Celso. Proliferation dynamics of acute myeloid leukaemia and haematopoietic progenitors competing for bone marrow space. Nature Communications, 9(1):519, February 2018.
